# Keggin-type polyoxotungstates as mushroom tyrosinase inhibitors - A speciation study

**DOI:** 10.1038/s41598-019-41261-7

**Published:** 2019-03-26

**Authors:** Joscha Breibeck, Nadiia I. Gumerova, Benedikt B. Boesen, Mathea Sophia Galanski, Annette Rompel

**Affiliations:** 1 Universität Wien, Fakultät für Chemie, Institut für Biophysikalische Chemie, Althanstraße 14, 1090 Wien, Austria; 2 Universität Wien, Fakultät für Chemie, Institut für Anorganische Chemie, Währinger Str. 42, 1090 Wien, Austria

**Keywords:** Metalloproteins, Metalloproteins

## Abstract

Mushroom tyrosinase *ab*PPO4 is a commercially relevant polyphenol oxidase and has been being targeted for numerous inhibition studies including polyoxometalates (POMs). In the present work, its diphenolase activity was inhibited at pH 6.8 by a series of structurally related polyoxotungstates (POTs) of the α-Keggin archetype, exhibiting the general formula [X^n+^W_12_O_40_]^(8−n)−^ in order to elucidate charge-dependent activity correlations. Kinetic data were obtained from the dopachrome assay and ^183^W NMR was applied to obtain crucial insights into the actual Keggin POT speciation in solution, facilitating a straightforward assignment of inhibition effects to the identified POT species. While [PW_12_O_40_]^3−^ was completely hydrolyzed to its moderately active lacunary form H_x_[PW_11_O_39_]^(7−x)−^ (*K*_*i*_ = 25.6 mM), [SiW_12_O_40_]^4−^ showed the most pronounced inhibition effects with a *K*_*i*_ of 4.7 mM despite of partial hydrolysis to its ineffective lacunary form H_x_[SiW_11_O_39_]^(8−x)−^. More negative Keggin cluster charges of 5− and 6− generally resulted in preclusion of inhibitory efficacy as well as hydrolysis, but with the Ni-substituted cluster [PW_11_O_39_{Ni(H_2_O)}]^5−^ enzymatic inhibition was clearly restored (*K*_*i*_ = 9.7 mM). The inhibitory capacity of the structurally intact Keggin POTs was found to be inversely correlated to their net charge. The here applied speciation strategy is of utmost importance for any biological POM application to identify the actually active POM species.

## Introduction

Polyphenol oxidases (PPOs) constitute an important family of enzymes occurring throughout the eukaryotic and prokaryotic kingdoms^1–3^. The PPO subgroup of catechol oxidases exhibits only diphenolase activity (EC 1.10.3.1, two-electron oxidation of *ortho*-diphenols to *ortho*-quinones), whereas the family of tyrosinases is capable of both monophenolase (EC 1.14.18.1, *ortho*-hydroxylation of monophenols to *ortho*-diphenols) and diphenolase activities^4^. PPOs share a common active site architecture comprising a type III dinuclear copper center in tight coordination by six conserved histidine residues. As the active site of PPOs is located on the protein surface^5^, it is well accessible for substrates and inhibitors. Among other substrate-related compounds, the most potent inhibitors are hydroquinone^6^, tropolone^7^ and kojic acid^8^ (C_6_H_6_O_4_, cf. Fig. S17 for structure), binding to the active site with inhibition constants (*K*_*i*_) in the µM range. In this series, kojic acid is the strongest and best characterized inhibitor, which is known to act in a competitive inhibition mode e.g. for “mushroom tyrosinase”^8^ or a mixed-type inhibition mode e.g. for *Bacillus megaterium* tyrosinase^6^. For its negligible lag phase and generally faster reaction velocity, the diphenolase activity of tyrosinases is usually assayed in kinetic studies. Importantly, the inhibition mode still depends on the substrate^9^, for which *L*-DOPA is most often reported in PPO inhibition studies, including those with kojic acid cited above.

Tyrosinase PPO from *Agaricus bisporus* (*ab*PPO) is predominantly used in PPO applications due to its high commercial availability as an enzymatic extract (“mushroom tyrosinase”) from mushroom fruiting bodies. Recently, a protocol for recombinant expression of *ab*PPO4 in *Escherichia coli* and purification to homogeneity was established^10^, which was followed to prepare pure *ab*PPO4 for this study. *Ab*PPO4 has been thoroughly investigated and crystallized after purification both from the natural source^11–13^ and after recombinant expression^10^, and therefore it is one of the few enzymes with confirmed identity for its native and recombinant form.

Polyoxometalates (POMs) are negatively charged metal oxide clusters built up from interconnected polyhedra^14^. For their versatility in size, charge and shape as well as a wide range of possible modifications, POMs have been receiving considerable attention for biological application in the past decades, including enzyme inhibition^15,16^, cytotoxicity studies investigating antibacterial^17^ and anti-tumour activity^18^ and even protein crystallization^19,20^. POMs have been recognized for their versatile binding behaviour towards biomolecules, especially proteins, driven by polar and non-polar interactions.

From the principal POM archetypes, the Keggin structure [X^n+^M_12_O_40_]^(8−n)−^ was chosen for this study. It comprises a relatively small spherical structure (ca. 10.4 Å in diameter) with well-established synthetic protocols, but at the same time remarkable compositional versatility. Thus, it is an ideal candidate for systematic variation of structural parameters. The Keggin archetype can be envisaged as an {M_12_O_36_} cage (addenda atom M: Mo(*VI*) or W(*VI*)) with zero net charge encapsulating an [X^n+^O_4_]^(8−n)−^ tetrahedron (central atom X: main group element or early transition element)^21^. Therein, each addenda atom is linked to 6 oxygen atoms (one terminal O_t_ and four bridging O_µ_ from M-O-M groups and 1 O_µ_ from X-O-M) resulting in a distorted octahedral geometry. The most stable Keggin isoform is the α-isomer with tetrahedral symmetry, and the {M_12_O_36_} shell can be grouped into four {M_3_O_13_} triads. Rotation of one, two, three or all four triads about 60° leads to the energetically less favored β-, γ-, δ- and ε-isomers^22^ (cf. Fig. S3).

The inhibition capacity of a few Keggin POMs against the enzymatic activity of PPOs has been addressed before in non-coherent and incommensurable studies investigating the polyanions in their acid forms H_3_[P^V^W_12_O_40_], H_4_[Si^IV^W_12_O_40_]^23^ or with glycinium countercations (HGly)_3_[P^V^W_12_O_40_], (HGly)_4_[Si^IV^W_12_O_40_]^24^ (Gly = glycine), or the monosubstituted Keggin derivatives with proposed formulas Na_7_[P^V^Mo_11_Cu^I^O_40_]^25^, Na_8_[Si^IV^W_11_Co^II^O_40_]^26^ and Na_6_[P^V^Mo_11_Fe^II^O_40_]^27^. However, each of these investigations was limited to only one or two different compounds, and parameters such as counterions, cluster charge and addenda atom substitution were varied in an arbitrary way, thereby obscuring possible trends in the structure-activity relationship of the applied POMs. In contradiction to their structural analogy, for (HGly)_3_[PW_12_O_40_] and (HGly)_4_[SiW_12_O_40_], completely different inhibition types and highly differing inhibitory capacities were determined^24^. The same discrepancies were reported for the two Keggin-POMs substituted with Cu^I ^^25^ or Fe^II ^^27^.

The Keggin clusters applied in this study were selected for the criteria of exhaustive structural verification, good synthetic accessibility and sufficient stability in aqueous solution. We entirely focussed on the α-Keggin isomer which can be prepared with a large number of different heteroatoms and shows unique structural stability even upon reversible reduction with up to 18 electrons^28^.

Seven different α-Keggin polyoxotungstates (POTs) were synthesized according to published procedures, with net charges in the range from 6− to 3−, and ensured high aqueous solubility with Na^+^ or K^+^ counterions. W was chosen over Mo due to its remarkable redox stability. The tested Keggin POTs (summarized in Fig. S2 and Table S1) were Na_3_[PW_12_O_40_]^21^ (abbreviated: **[PW**_**12**_**]**^**3−**^), K_4_[SiW_12_O_40_]^29^ (**[SiW**_**12**_**]**^**4−**^), K_5_[BW_12_O_40_]^30^ (**[BW**_**12**_**]**^**5−**^), Na_5_[AlW_12_O_40_]^31^ (**[AlW**_**12**_**]**^**5−**^), Na_6_[H_2_W_12_O_40_]^32^ (**[H**_**2**_**W**_**12**_**]**^**6−**^), Na_6_[BeW_12_O_40_]^33^ (**[BeW**_**12**_**]**^**6−**^), K_5_[PW_11_O_39_{Ni(H_2_O)}]^34^ (**[PW**_**11**_**Ni]**^**5−**^). The lacunary anions K_7_[PW_11_O_39_]^35^, K_8_[SiW_11_O_39_]^36^ and Na_9_[AlW_11_O_39_]^31^ were prepared as control compounds to account for the potential hydrolysis of the corresponding Keggin POTs. Metatungstate **[H**_**2**_**W**_**12**_**]**^**6−**^ is the most common Keggin cluster with net charge 6−. As an isopolytungstate, it lacks the typical central atom with tetrahedral coordination and carries two protons instead, therefore showing a slightly distorted structure with respect to the other Keggin clusters and a questionable hydrolytic stability at pH > 6.4^37^. For comparison, we investigated **[BeW**_**12**_**]**^**6−**^ with higher hydrolytic stability up to pH 9.5. In the substituted Keggin anion (**[PW**_**11**_**Ni]**^**5−**^, one of the W(*VI*) addenda atoms is exchanged by Ni(*II*) in order to possibly tune the POT’s protein affinity. The coordination sphere of the light transition metal is completed by one labile water ligand (cf. Fig. S2), which can be easily replaced by nucleophilic amino acid side chains on a protein surface to form a direct bond with the POT. The low oxidation state of Ni(*II*) results in a final cluster net charge of 5−, which is well comparable to **[BW**_**12**_**]**^**5−**^ and **[AlW**_**12**_**]**^**5−**^. Anions with central atoms comprising an unusually low charge (+1 in [CuW_12_O_40_]^7− ^^38^) have been reported only in solution and characterized as a transient oxidation state highly sensitive to re-oxidation. Clusters with extraordinarily highly charged central atoms (+6 in [SW_12_O_40_]^2− ^^39^ and [SeW_12_O_40_]^2− ^^40^) have been obtained exclusively from non-aqueous media as counter anions for other metal complexes supporting their structural framework. Therefore they were excluded from our study due to doubtful synthetic accessibility and stability in aqueous solution.

The hydrolytic stability of Keggin POT anions correlates with their net charge, and one single additional negative charge can increase the cluster stability by 2 to 3 pH units^14,33^ (Fig. S2). Therefore, the POTs with highest negative charge like **[BeW**_**12**_**]**^**6−**^ are expected to be most stable in aqueous buffers up to pH 9, followed by **[BW**_**12**_**]**^**5−**^ and **[AlW**_**12**_**]**^**5−**^ with stability up to pH 6.5 and **[SiW**_**12**_**]**^**4−**^ up to pH 4.5. The most labile compound in this respect is **[PW**_**12**_**]**^**3−**^ carrying the lowest negative charge with stability up to pH 1.5. Potential POT hydrolysis is most likely triggered by the attack of hydroxide anions and prevented with increasing electrostatic repulsion from the negatively charged cluster surface^14^. Apparently, in a physiological pH range as required for optimal activity of *ab*PPO4 and for most other biological investigations, the low charge clusters **[PW**_**12**_**]**^**3−**^ and **[SiW**_**12**_**]**^**4−**^ should not be considered as intact species and have to be expected to undergo at least partial hydrolysis. Surprisingly, probably due to good synthetic accessibility, it is these two (in this regard most unstable) Keggin anions that most biological applications have focussed on up to now (e.g.^15,24,41,42^), without careful speciation of POM clusters under the applied physiological buffer conditions. Here, we present the first systematic investigation of *ab*PPO4 inhibition towards its diphenolase activity through POT Keggin anions, including the careful speciation of respective POT clusters at physiological pH 6.8 by ^183^W-NMR and ESI-MS analysis to address cluster species responsible for interaction with the enzyme in solution. Thereby, we intended to derive a possible correlation between Keggin POT properties and the observed inhibitory effect against *ab*PPO4 diphenolase activity, focussing on the overall surface charge conveyed by different central atoms as a systematically varied parameter.

## Results and Discussion

### Activity plots of abPPO4 inhibited by Keggin POTs

*Ab*PPO4 was prepared in its activated state according to the published procedure by Pretzler *et al*.^10^. (Fig. S1). All Keggin POT clusters were synthesized following published protocols (see Table S1) and verified by IR spectroscopy (Figs S4, S5) prior to further investigation. As a positive control for the inhibition of *ab*PPO4 diphenolase activity and as a validation of the kinetic methodology, all kinetic experiments described here were additionally performed with the well-characterized natural PPO-inhibitor kojic acid and evaluated applying exactly the same mathematical model.

The detailed methodology applied for kinetic evaluation is presented in the SI. For systematic investigation of the potential inhibition of *ab*PPO4 by different Keggin clusters, the dopachrome assay was performed at a fixed reference substrate concentration of 1 mM *L*-DOPA and in a concentration range of 0–14 mM POT. If enzymatic inhibition was observed, data were fit by a hyperbolic expression derived from the mixed inhibition model (SI equation (6)), which accounted for all potential inhibition types and allowed evaluation of inhibition constants *K*_*i*_ and *α*-parameters (summarized in Table 1).

**Table 1:** Combined kinetic evaluation of *ab*PPO4 inhibition by Keggin POTs with controls kojic acid, Na_2_WO_4_ and Ni(NO_3_)_2_. *K*_*i*_, inhibition constant; α, inhibition parameter; R^2^, curve fit determination coefficient.

Effector	*K*_*i*_ [mM]	*α*	R^2^	Inhibition type
**[PW**_**12**_ **]** ^**3−**^	25.6^[a]^, 18.6^[b]^, 20.1^[c]^	0.13^[a]^, 0.20^[b]^	0.98	Mixed-type
**[SiW**_**12**_ **]** ^**4−**^	4.7^[a]^, 3.8^[b]^, 5.6^[c]^	0.11^[a]^, 0.32^[b]^	0.92	Mixed-type
**[BW**_**12**_ **]** ^**5−**^	No inhibition			
**[AlW**_**12**_ **]** ^**5−**^	No inhibition			
**[H**_**2**_ **W**_**12**_ **]** ^**6−**^	No inhibition			
**[BeW**_**12**_ **]** ^**6−**^	No inhibition			
**[PW**_**11**_ **Ni]** ^**5−**^	9.7^[a]^, 14.3^[b]^, 15.0^[c]^	0.12^[a]^, 0.20^[b]^	0.98	Mixed-type
Control: **[WO**_**4**_**]**^**2−**^	20.0^[a]^, 19.0^[b]^, 24.8^[c]^	0.14^[a]^, 0.03^[b]^	0.97	Mixed-type
Control: **[PW**_**11**_**]**^**7−**^	12.0^[a]^, 9.9^[b]^, 9.9^[c]^	0.12^[a]^, 0.17^[b]^	0.91	Mixed-type
Control: **[SiW**_**11**_**]**^**8−**^	No inhibition			
Control: **[AlW**_**11**_**]**^**9−**^	54.1^[a]^, 38.8^[b]^, 45.7^[c]^	0.12^[a]^, 0.31^[b]^	0.89	Mixed-type
Control: Ni^2+^	No inhibition			
Control: kojic acid	5.1^[a]^, 4.5^[b]^, 4.3^[c]^ µM	2.6•10^15^	1.0	Competitive

^[a]^from activity plot, ^[b]^from Lineweaver-Burk slopes or intercepts, ^[c]^from Dixon plots.

**Figure 1:**
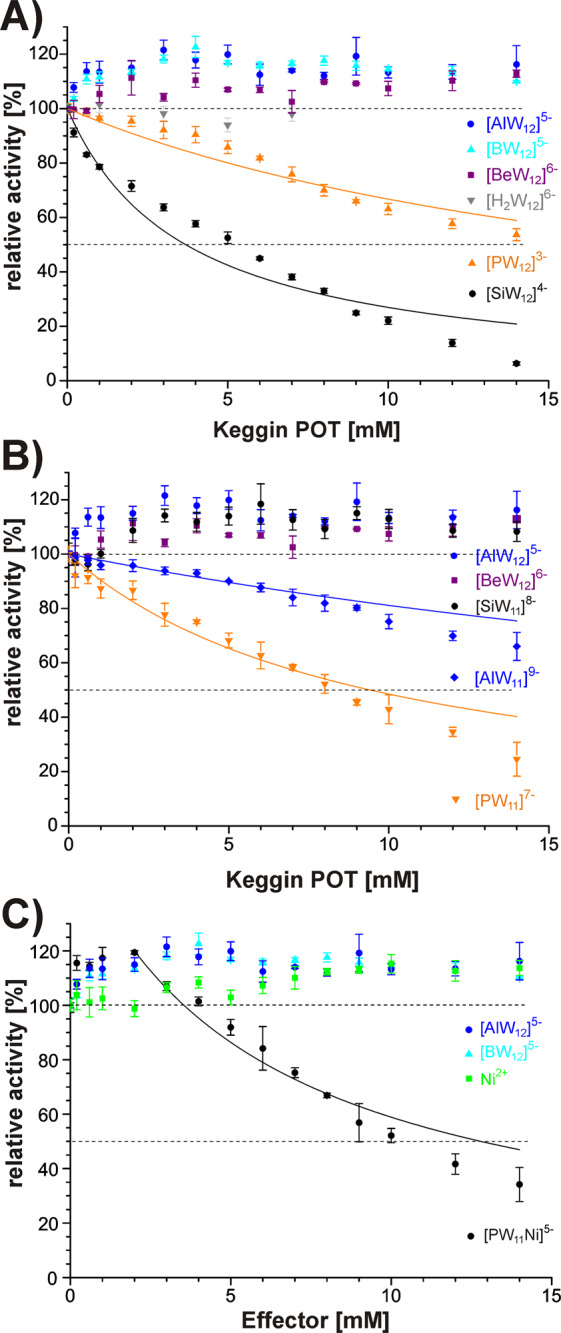
Activity plots of *ab*PPO4 (1 µg) with (**A**) various non-substituted Keggin clusters of different charge, (**B**) lacunary anions [XW_11_]^n−^ and intact Keggin POTs of comparable charge state in buffered solution, and (**C**) with Keggin clusters of a common charge 5− and control with Ni(NO_3_)_2_. The dopachrome assay was performed with 1 mM *L*-DOPA (in 50 mM Na-citrate pH 6.8) and 0–14 mM POTs. The initial linear reaction rates were normalized with respect to the non-inhibited reaction velocity to be plotted as relative enzymatic activities and fitted according to equation (6) (see SI). For fit parameters, see Table 1.

Kojic acid behaved as an ideal competitive inhibitor of *ab*PPO4, as strongly proposed by the large *α*-parameter determined from the perfect activity curve fit (Fig. S17) and consistent with data in the literature^8^. The applied curve fit (Fig. 1) describes the POTs inhibition behaviour well enough to provide valuable quantitative estimates for kinetic parameters, in line with the other analyses presented later on, albeit the POT interaction might comprise additional binding phases to different positions at the *ab*PPO4 surface.

Among the Keggin clusters without addenda atom substitution, **[SiW**_**12**_**]**^**4−**^ clearly showed the most potent inhibitory effect (Fig. 1A), and the fitted *α*-parameter suggests a mixed mode of inhibition (cf. Table 1). The other inhibiting compound **[PW**_**12**_**]**^**3−**^ with one less negative charge exhibited a markedly lower inhibition capacity, suggesting that the more negatively charged clusters would perform best. Interestingly, increasing the net charge up to 5− and even 6− completely obliterated inhibition of *ab*PPO4 activity and even effected a concentration-independent enzyme activation about 20%.

A possible explanation for these observations would be an allosteric mechanism of action on the enzyme. According to the assigned mixed-type of inhibition, **[PW**_**12**_**]**^**3−**^ and **[SiW**_**12**_**]**^**4−**^ are likely to bind to both the free enzyme and the enzyme-substrate complex, but at protein sites distinct from the substrate binding pocket, altering the enzyme conformation towards decreased activity. The POTs with higher charges of 5− and 6− could act in a similar way, but trap the enzyme in a conformation which is better accessible to the substrate than the native state, resulting in the observed rise in activity.

### AbPPO4 inhibition by lacunary Keggin POTs

Control studies were performed with pure lacunary anions of the two active Keggin POTs as well as the non-inhibiting cluster **[AlW**_**12**_**]**^**5−**^ in order to elucidate a similar surface charge tendency as obtained with the intact anions. In spite of its high formal charge, **[PW**_**11**_**]**^**7−**^ featured an inhibitory effect of intermediate capacity (Fig. 1B) between the two active intact POTs, whereas **[SiW**_**11**_**]**^**8−**^ showed no enzymatic inhibition and behaved similar to the highly charged intact Keggin anions. In contrast to its even higher negative charge, **[AlW**_**11**_**]**^**9−**^ exhibited a weak inhibitory capacity, hinting to a structural rearrangement towards another active species.

### AbPPO4 inhibition by a Ni-substituted Keggin anion

The monosubstituted **[PW**_**11**_**Ni]**^**5−**^ cluster (cf. Figs S8, S15, S16, Table S2) effected pronounced inhibition of *ab*PPO4 in the higher concentration ranges (Fig. 1C), probably due to specific protein-metal interaction, featuring a superior inhibitory capacity with regard to analogous non-substituted compounds with charge 5− which showed no such effects at all. In a similar way as observed for its charge analogues, **[PW**_**11**_**Ni]**^**5−**^ led to a slight increase in the apparent reaction rates at low concentrations, which was however quickly outweighed by the inhibitory capacity of this compound. Potential hydrolysis of the substituted Keggin anion should firstly yield the non-inhibiting lacunary form [PW_11_O_39_]^7−^ and Ni^2+^ ions, which is why control experiments were performed with a nickel(*II*) salt (Fig. 1B), revealing no inhibition effect and excluding free Ni^2+^ ions as the inhibiting species.

### AbPPO4 inhibition by orthotungstate control

**Figure 2:**
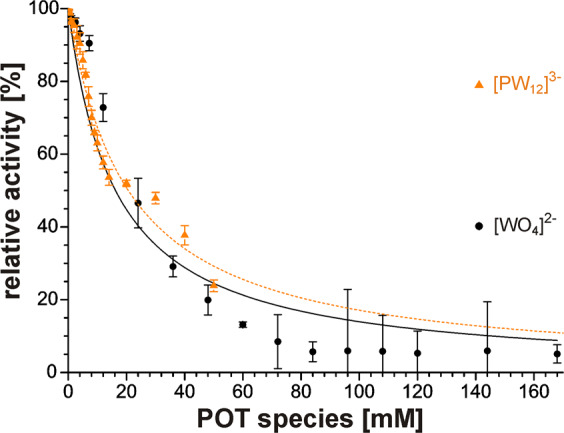
Activity plots of *ab*PPO4 (1 µg) with **[PW**_**12**_**]**^**3−**^ and **[WO**_**4**_**]**^**2−**^. The dopachrome assay was performed with 1 mM *L*-DOPA (in 50 mM Na-citrate pH 6.8) and 0–14 mM POTs. The initial linear reaction rates were normalized with respect to the non-inhibited reaction velocity to be plotted as relative enzymatic activities and fitted according to equation (6) (see SI). For fit parameters, see Table 1.

Keeping in mind the potential hydrolysis of Keggin clusters with lower charge, control experiments with free orthotungstate **[WO**_**4**_**]**^**2−**^ have been included. To account for the overall tungsten amount present in 0–14 mM [XW_12_]^n−^ Keggin POT, the concentration range was even extended to its twelvefold excess (0–168 mM **[WO**_**4**_**]**^**2−**^). The strongly basic Na_2_[WO_4_] stock solution was adjusted for pH 6.8 with HCl. The resulting activity plot (Fig. 2) was in striking accordance with the plot for **[PW**_**12**_**]**^**3−**^, suggesting that the observed inhibition might not exclusively originate from the Keggin cluster, but from other polytungstate species present in both samples. Still, all the determined *α*-parameters were in good agreement, indicating a similar inhibition behaviour for the structurally related tungsten compounds.

### Determination of inhibition modes for effective compounds

The type of enzymatic inhibition is commonly investigated by linear plots according to Lineweaver-Burk^43^ and Dixon^44^, also allowing for further validation of the inhibition constant *K*_*i*_. In this respect, for each of the inhibiting compounds (kojic acid control, **[WO**_**4**_**]**^**2−**^ control, **[PW**_**12**_**]**^**3−**^, **[SiW**_**12**_**]**^**4−**^, **[PW**_**11**_**Ni]**^**5−**^) the dopachrome assay was repeated at five different substrate concentrations and three different inhibitor concentrations, respectively (SI section 3.2).

### Lineweaver-Burk evaluation of inhibition types

For the kojic acid control, the Lineweaver-Burk lines (Fig. S18A) converged on the ordinate (corresponding to a common maximum reaction rate), clearly supporting the competitive inhibition mode for this compound. The Dixon plots (Fig. S18B) additionally confirmed these findings with a common intersection point at −*K*_*i*_ on the abscissa, as found for competitive inhibitors. The obtained *K*_*i*_ values of about 4.5 µM perfectly matched the data reported for kojic acid^8^, thus validating the here performed kinetic evaluations as a reliable experimental approach.

**Figure 3:**
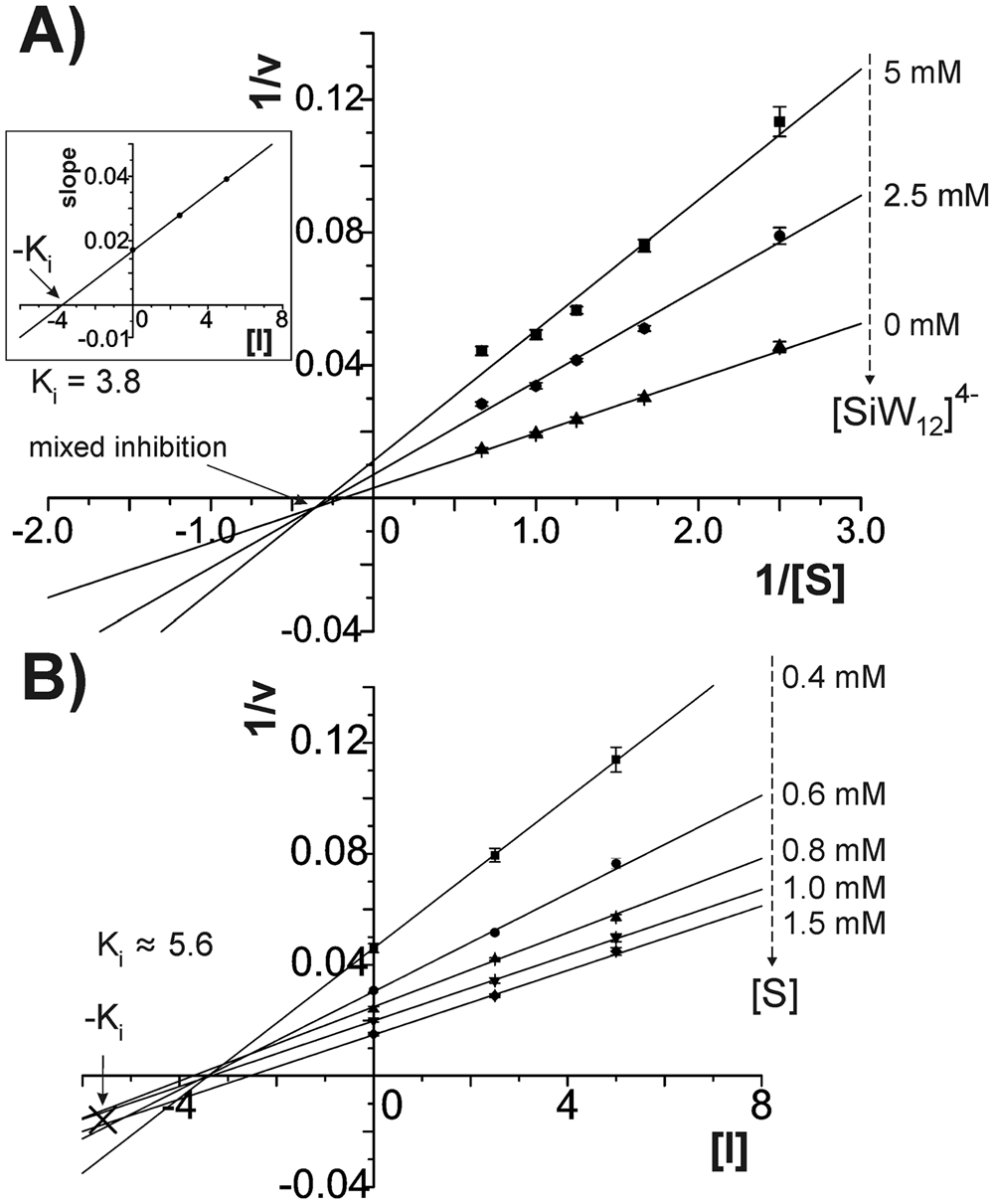
Kinetic evaluation of *ab*PPO4 inhibition by **[SiW**_**12**_**]**^**4−**^ using (**A**) Lineweaver-Burk plots and (**B**) Dixon plots. (**A**) The intersection behaviour of the fitted straight lines (SI equation (7)) suggests mixed-type inhibition for the Keggin POT. Inset: Plotting the linear slopes against POT concentration allows for an estimate of *K*_*i*_ (SI equation (8)). (**B**) The average intersection of the Dixon plots yields a third *K*_*i*_ estimate (SI equation (10)). For fit parameters, see Table S3.

With the polytungstates, the Lineweaver-Burk plots (Figs 3A, S19–S23A) of the resulting reaction velocities yielded three lines (one for each inhibitor concentration) with a common intersection point. Plotting the slopes of the Lineweaver-Burk lines against the applied inhibitor concentration (insets in Figs 3A, S19–S23A) resulted in straight lines intersecting the abscissa at −*K*_*i*_, thus a revalidation of the *K*_*i*_ value determined from the activity plots before (cf. Table 1). In a similar fashion, the ordinate intercepts of the Lineweaver-Burk lines were evaluated for the *α*-parameter (cf. Tables S4 and 1). The inhibiting Keggin compounds showed common features, as can be expected as a consequence of their structural similarity. The Lineweaver-Burk interception points were found in the third quadrant of the coordinate system, which is commonly observed for mixed-type inhibitors^45^.

### Confirmation of inhibition types by Dixon plots

The Dixon plots (Fig. 3B, S19–S23B) were used to obtain a third estimate of the *K*_*i*_ value from five lines (one for each substrate concentration). The Dixon lines did not intersect in one common point when fitted to straight lines, which can be explained by the mixed-type behaviour^44^, but still allowed for *K*_*i*_ estimation as the average of all intersection points between the lines. The *K*_*i*_ values extracted by three different methods (cf. Table 1) were in good accordance for all investigated compounds, thus supporting the consistency of the presented results.

### Speciation of Keggin POTs at pH 6.8 by NMR analyses

In biological application studies employing POMs so far, the Keggin compounds were only verified by UV-Vis and IR spectroscopy, which cannot provide the detailed structural information required for speciation under the respective experimental conditions.

**Figure 4:**
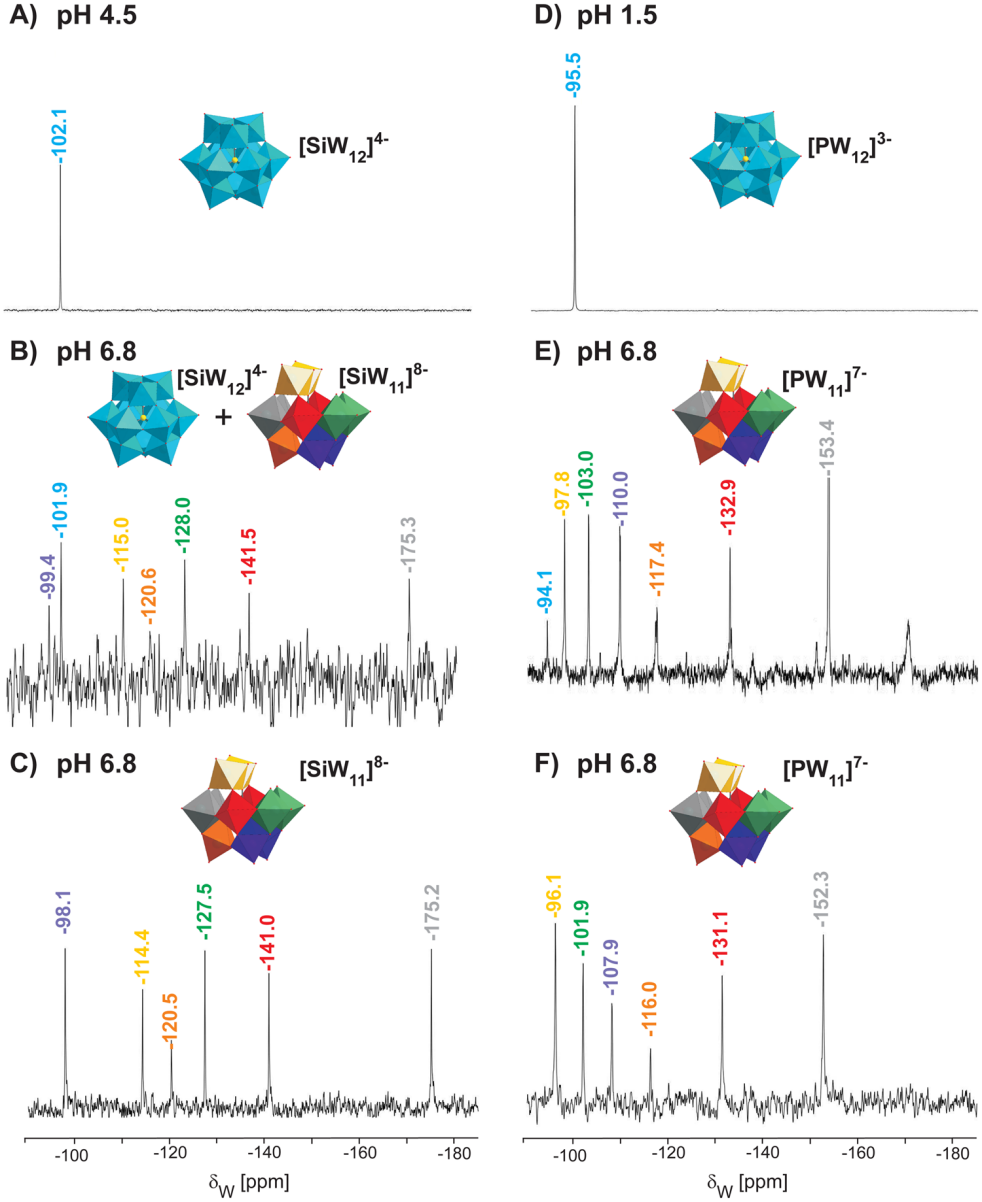
^183^W-NMR spectra of **[SiW**_**12**_**]**^**4−**^ and **[PW**_**12**_**]**^**3−**^. (**A**) **[SiW**_**12**_**]**^**4−**^ in 50 mM NaOAc pH 4.5; (**B**) **[SiW**_**12**_**]**^**4−**^ at pH 6.8; (**C**) **[SiW**_**11**_**]**^**8−**^ at pH 6.8, signal assignment according to^46^; (**D**) **[PW**_**12**_**]**^**3−**^ at pH 1.5; (**E**) **[PW**_**12**_**]**^**3−**^ at pH 6.8, (**F**) **[PW**_**11**_**]**^**7−**^ at pH 6.8, signal assignment according to^49,50^. Partial hydrolysis of both Keggin clusters at pH 6.8 is clearly evident. Keggin POTs were dissolved in buffer to obtain 60 mg/mL solutions (approximately 20 mM). The total recording time is about 60 hours for all measurements, chemical shifts were measured relative to external 1 M Na_2_WO_4_. Color code for intact cluster: {WO_6_}, blue; P or Si, yellow; O, red. The NMR signals are assigned to W nuclei sharing the same chemical environment according to literature data.

^183^W NMR spectroscopy is the most widely applied and reliable technique for the structural characterization of POTs in solution as well as to monitor the progress of reaction^46^. Hundreds of POM ^183^W NMR spectra have been reported since the first ^183^W NMR spectrum for **[SiW**_**11**_**]**^**8−**^ observed by Acerete *et al*. in^47^. A timeline summarizing the structural characterization of Keggin POTs most relevant to this study is given in Fig. S25. The here-presented ^183^W NMR analysis yielded a single signal for all intact [XW_12_]^n−^ structures (Figs 4A,D and S11–S14), reflecting the perfect tetrahedral symmetry of the Keggin α-isomer and clearly confirming the isomeric purity of the prepared POTs. The Keggin POTs with higher charges **[BW**_**12**_**]**^**5−**^, **[AlW**_**12**_**]**^**5−**^ (cf. Fig. S6), **[H**_**2**_**W**_**12**_**]**^**6−**^, **[BeW**_**12**_**]**^**6−**^ (cf. Fig. S9) were thereby verified to retain full structural integrity at pH 6.8. Symmetry breaking by isomerisation, decomposition or substitution leads to an increase in the number of NMR signals with defined shifts and relative intensities and allows for structural assignment and speciation of clusters such as the mono-substituted Keggin structure]^48^. ^183^W NMR measurements of the **[SiW**_**12**_**]**^**4−**^ cluster at pH 4.5 (Fig. 4A) and 6.8 (Fig. 4B) revealed that partial hydrolysis to the lacunary form **[SiW**_**11**_**]**^**8−**^ reduced the amount of intact POT in solution.

### Hydrolytic stability of Keggin POTs at pH 6.8

Upon dissolution in reaction buffer (50 mM Na-citrate pH 6.8) for preparation of a 20 mM POT stock, Na_3_[PW_12_O_40_] showed a strongly acidic reaction (final pH of 3.5). For the sake of comparison, the pH of the **[PW**_**12**_**]**^**3−**^ stock solution was re-adjusted to 6.8 prior to enzymatic measurements. The ^183^W NMR analysis of the **[PW**_**12**_**]**^**3−**^ cluster at pH 1.5 (Fig. 4D) and 6.8 (Fig. 4E) unambiguously confirmed hydrolysis to its lacunary form [PW_11_O_39_]^7−^ ^45,47,49,50^ and several other polytungstate species, as also supported by additional ^31^P NMR data^51^ (Fig. S7). The preparations of pure lacunary clusters **[SiW**_**11**_**]**^**8−**^ (Fig. 4C) and **[PW**_**11**_**]**^**7−**^ (Fig. 4F) served as a control compounds in the NMR analyses. The higher the charge of the POT anions, the more basic was their reaction when dissolved in buffer pH 6.8, and solution pH had to be adjusted by HCl addition.

For [PW_12_O_40_]^3−^, hydrolytic stability at different pH values has been addressed before by ^31^P NMR spectroscopy and ESI mass spectrometry, with the clear result that the lacunary cluster [PW_11_O_39_]^7−^ is the only prevailing species in the neutral pH range^51–53^, which is in perfect agreement with the NMR-based speciation in this study.

Therefore, the apparent inhibitory capacities at pH 6.8 in the buffer system applied for the kinetic measurements here can be interpreted as the combined effects of various tungstate species in solution. Decomposition of the intact Keggin cluster to the lacunary species according to (1) and further equilibration to a complex phosphotungstates mixture (not shown in eq. (1)) consumes a high amount of hydroxide anions, thereby lowering the pH level, and is a reasonable explanation for the observations.

1 $${[{{\rm{P}}{\rm{W}}}_{12}{{\rm{O}}}_{40}]}^{3-}+{2{\rm{O}}{\rm{H}}}^{-}+{{\rm{H}}}_{2}{\rm{O}}\leftrightarrows {{\rm{H}}}_{3}{[{{\rm{P}}{\rm{W}}}_{11}{{\rm{O}}}_{39}]}^{4-}+{\rm{H}}{[{{\rm{W}}{\rm{O}}}_{4}]}^{-}$$

### Charge dependence of inhibitory effects of intact and lacunary Keggin POT species

Based on the observed lack of inhibitory capacity exhibited by highly charged Keggin POMs such as **[BW**_**12**_**]**^**5−**^ or **[H**_**2**_**W**_**12**_**]**^**6−**^, the lacunary anions [SiW_11_O_39_]^8−^ and [PW_11_O_39_]^7−^ were not expected to contribute to the observed inhibitory effects. However, the oxygen atoms surrounding the lacuna are much more basic than the bridging and terminal atoms and most likely protonated to a certain extent^54^. In neutral aqueous solution, binding of up to three protons was suggested for [SiW_11_O_39_]^8−^ and confirmed by conductivity measurements for [PW_11_O_39_]^7−^, resulting in the predominant species H_3_[PW_11_O_39_]^4−^ ^55^. This POT species with a surface charge distribution similar to [SiW_12_O_40_]^4−^ is therefore responsible for most of the inhibitory capacity found for the **[PW**_**12**_**]**^**3−**^ and **[PW**_**11**_**]**^**7−**^ setups. H_3_[SiW_11_O_39_]^5−^ or less protonated species comprise too high charge densities for inhibitory interaction with *ab*PPO4, in line with the intact POTs with charges 5− and 6−. The solution speciations of **[PW**_**12**_**]**^**3−**^ and **[SiW**_**12**_**]**^**4−**^ indicate the possibility that even stronger inhibition could be achieved if full integrity of these Keggin POTs were maintainable at neutral pH. The lacunary Keggin **[AlW**_**11**_**]**^**9−**^ prepared for control experiments was not stable at pH 6.8 and rearranged to the monosubstituted structure [AlW_11_O_39_{Al(H_2_O)}]^6−^, which was revealed by ^27^Al NMR (Fig. S6C) and ^183^W analyses (Fig. S12B). Featuring a potential coordination site in analogy to **[PW**_**11**_**Ni]**^**5−**^ at its surface-exposed aluminum(*III*) atom, this compound exhibited weak inhibition effects despite the overall high charge.

Interestingly, ^183^W NMR speciation of the **[WO**_**4**_**]**^**2−**^ solution at pH 6.8 revealed the heptatungstate anion H[W_7_O_24_]^5−^ ^56^ as the only present species to evoke the exhibited inhibitory effect (Fig. S10), with no detectable residual free orthotungstate. Despite its high overall charge of 5− which prevented enzymatic inhibition by Keggin POTs, heptatungstate appears to have a unique favourable charge distribution suitable for *ab*PPO4 interaction. The here-presented data suggests small polytungstate species such as H[W_7_O_24_]^5−^ to contribute to the moderate inhibitory capacity of **[PW**_**12**_**]**^**3−**^ at pH 6.8, which was lower than measured for the pure lacunary anion **[PW**_**11**_**]**^**7−**^ and reflects the inhomogeneous composition detected by ^31^P NMR data (Fig. S7B).

## Conclusions

**Figure 5:**
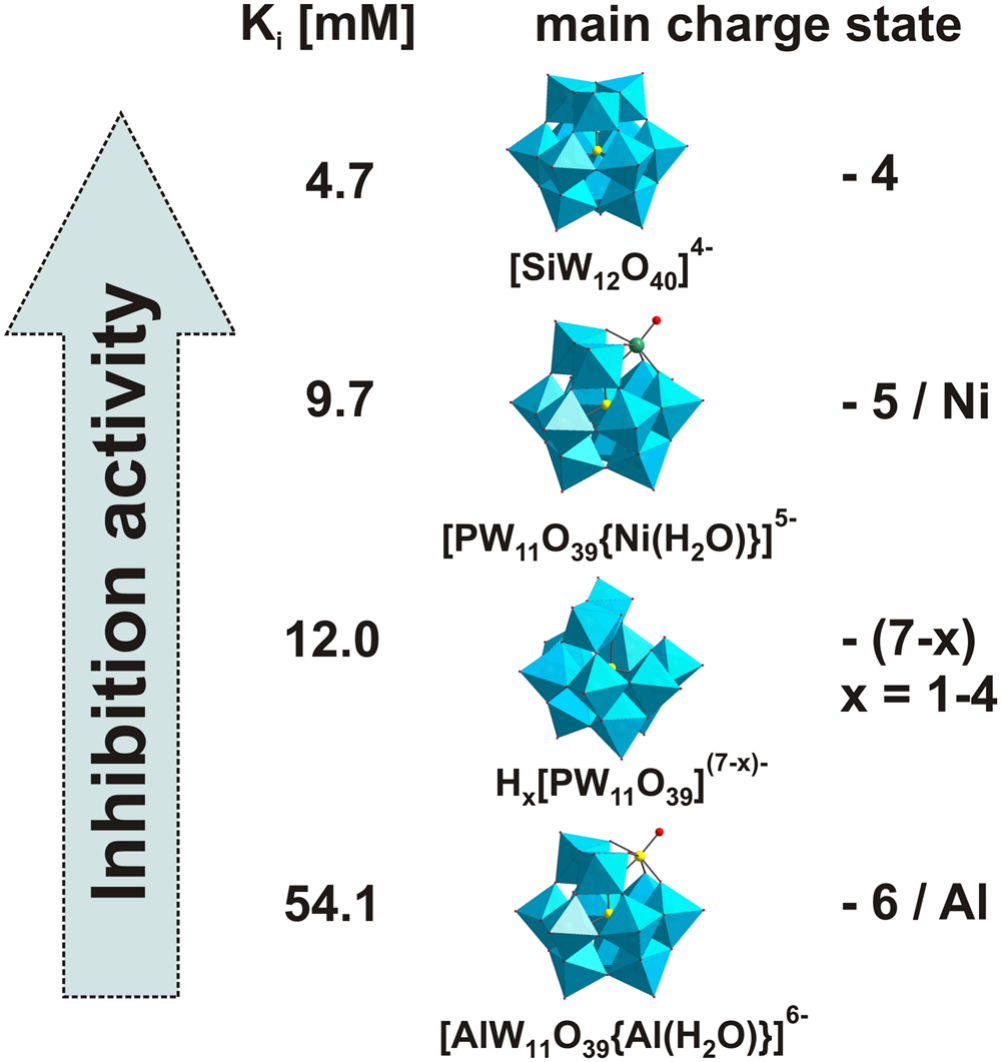
Comparison of *ab*PPO4 inhibition capacities of all identified Keggin POT clusters. Inhibitory activities were only observed for non-substituted POT species with an overall negative charge of 4−. However, introduction of metal centers with available coordination sites to the cluster surface was found suitable to equip even more highly charged anions with moderate binding activities.

The charge-dependent enzymatic inhibition behaviour of the α-Keggin POT archetype against *ab*PPO4 was characterized (Fig. 5), revealing significant inhibitory effects for POTs with lower net charge (**[PW**_**12**_**]**^**3−**^ and **[SiW**_**12**_**]**^**4−**^) counterbalanced by their pronounced hydrolysis. Although the POT anions with higher charges 5− and 6− even effected an increase in enzymatic activity, inhibitory activity was restored with the cluster **[PW**_**11**_**Ni]**^**5−**^ equipped with a nickel center for increased protein affinity.

## Methods

All chemicals have been purchased from Sigma-Aldrich (Vienna, Austria) and Carl-Roth (Karlsruhe, Germany) and were at least of analytical grade. Synthetic details and spectroscopic data along with the kinetic curves are presented in SI.

### Preparation of abPPO4 in its active form

The recombinant enzyme was prepared according to the published procedure by Pretzler *et al*.^10^. Briefly, a pGEX vector (GE Healthcare Europe; Freiburg, Germany) was used to equip the *ab*PPO4 gene with an N-terminal glutathione-*S*-transferase tag. The encoded fusion protein was expressed in *E*. *coli* BL21(DE3) in the auto-inducing medium ZYM-5052 in 2 L shaking flasks. Cells were cultivated at 20 °C for approximately 20 h, supplemented with 0.5 mM CuSO_4_ and grown for 20 more hours. The cell pellet was harvested by centrifugation, resuspended in a Tris/HCl lysis buffer with protease inhibitors and EDTA and disrupted by homogenization through a French pressure cell. After clarification, the supernatant containing the fusion protein was applied to a GSTrap FF column (GE Healthcare Europe) and eluted in pure form with reduced glutathione, followed by digestion with recombinant HRV 3 C protease in order to cleave off the GST tag. A second purification step employing GSTrap yielded pure latent *ab*PPO4 enzyme in the flow-through. The latent enzyme was treated with Proteinase K to specifically remove the C-terminal part for permanent activation and purified by size-exclusion chromatography using a Tricorn Superdex 200 16/60 column (GE Healthcare) in reaction buffer (50 mM Na-citrate pH 6.8). The combined protein fractions were concentrated and used for the kinetic analyses presented here (for a summary of the protein purification, see Fig. S1).

### Synthesis of α-Keggin POTs

All chemicals were purchased from Sigma-Aldrich (Austria) and used without further purification. The Keggin type polytungstates were synthesized according to the reported procedures (Table S1), which were slightly modified with regard to the following steps:

1. Na_6_[BeW_12_O_40_]·6 H_2_O was obtained via a cation exchange reaction between (Bu_4_N)_4.8_Na_1.2_[BeW_12_O_40_]^33^ and NaClO_4_ in acetonitrile.
2. K_5_[BW_12_O_40_]·18 H_2_O was prepared according to^57^ and re-crystallized three times from water at 60 °C. Mono-crystalline needle-shaped crystals of K_5_[BW_12_O_40_]·18 H_2_O were identified by checking the unit cell parameters (hexagonal, *a* = 18.97 Å, *b* = 18.97 Å, *c* = 12.4 Å, *α* = 90°, β = 90°, γ = 120°) on a Bruker D8 Venture.
3. Na_3_[PW_12_O_40_]·3 H_2_O was used instead of H_3_[PW_12_O_40_] as a starting material for the synthesis of K_5_[PW_11_NiO_40_H_2_]·11 H_2_O^58^.

The exact amount of crystal water in all Keggin POTs was determined gravimetrically by calcination at 200 °C for 10 hours.

### IR spectroscopy

After structural verification by unit cell determination, K_5_[BW_12_O_40_] was additionally analyzed by IR measurements on a Bruker Vertex 70 IR Spectrometer equipped with a single-reflection diamond-ATR unit. The obtained spectrum shows the characteristic bands for the α-Keggin polyoxotungstate cluster^57^ and was used as a reference spectrum for all the other POT compounds yielding highly similar spectra (see Figs S4 and S5). The W-O-W vibrations appear in the range 400–900 cm^–1^, while the terminal W = O vibrations occur close to 930–950 cm^−1^.

### NMR spectroscopy

^183^W NMR samples for all the POM compounds were prepared in high concentrations similar to the stock solutions used for kinetic experiments (usually about 20 mM) in 50 mM Na-citrate pH 6.8 if not stated otherwise. ^183^W NMR spectra were recorded with a Bruker FT-NMR spectrometer Avance Neo 500 MHz in 10 mm tubes for a total experiment time of ca. 60 hours, using standard pulse programs at 20.836 MHz and a 63° flip angle with 1 s of relaxation delay; the temperature was kept at 25 °C. Chemical shifts were measured relative to external 1 M Na_2_WO_4_. For **[PW**_**12**_**]**^**3−**^, **[PW**_**11**_**]**^**7−**^ and **[PW**_**12**_**Ni]**^**5−**^, additional ^31^P NMR spectra were taken, **structures of [AlW**_**12**_**]**^**5−**^, **[AlW**_**11**_**]**^**9−**^ and **[BeW**_**12**_**]**^**6−**^ were confirmed by ^27^Al NMR or ^9^Be NMR spectroscopy, respectively. ^27^Al, ^9^Be and ^31^P NMR spectra were recorded at 130.368, 70.273 and 202.53 MHz, respectively. Chemical shifts are given relative to Al(NO_3_)_3_, BeSO_4_ or 85% H_3_PO_4_.

### ^27^Al NMR

During synthesis of **[AlW**_**12**_**]**^**5−**^, an aliquot was taken from the reaction solution and checked for the isomerisation state of the Keggin anion by ^27^Al NMR spectroscopy. The obtained spectrum (Fig. S6A) is dominated by two intense signals, with one at 72 ppm corresponding to tetrahedral Al in α-**[AlW**_**12**_**]**^**5−**^^59^ and the other one at 0 ppm to the octahedral aluminum cation, which also serves as the reference signal in ^27^Al-NMR. This analysis proves that exclusively the α-Keggin isomer was obtained in our synthesis of **[AlW**_**12**_**]**^**5−**^, as other isomers with reduced symmetry would have yielded more complex NMR spectra. Taken together with the highly similar IR spectra (Fig. S4) recorded for all Keggin clusters, the isomeric purity for all POM compounds in this study is demonstrated. Similarly to its parent Keggin cluster, the lacunary anion **[AlW**_**11**_**]**^**9−**^ was confirmed as a pure compound (Fig. S6B). When dissolved at 60 g/L in reaction buffer pH 6.8, the strongly basic anion led to a pH increase to 8.5. Reacidification to pH 6.8 resulted in rearrangement of the POT species in solution to yield the monosubstituted cluster [AlW_11_O_39_{Al(H_2_O)}]^6−^, abbreviated as **[AlW**_**11**_**Al]**^**6−**^, which is characterized by two signals at 8.7 and 74.6 ppm^31^. The additional signal at 72.0 ppm hinting towards the intact anion **[AlW**_**12**_**]**^**5−**^ is unexpected^31^, as the corresponding ^183^W NMR spectrum (Fig. S12B) unambiguously establishes **[AlW**_**11**_**Al]**^**6−**^ as the only polytungstate species in solution.

### ^31^P NMR

In the ^183^W NMR spectrum of **[PW**_**12**_**]**^**3−**^ at pH 6.8 (see Fig. 4E), the six most intense peaks can be assigned to six different symmetry-equivalent positions in **[PW**_**11**_**]**^**7−**^^47,49^. The less intense signals in the ^183^W NMR spectrum can be attributed to other **[PW**_**12**_**]**^**3−**^ hydrolysis products^51^ that have been identified in the ^31^P NMR spectrum (Fig. S7B). The absence of signals at 15 ppm in the ^31^P NMR spectrum (Fig. S7B) and at 95 ppm in the ^183^W spectrum (cf. Fig. 4D) indicates full hydrolysis of **[PW**_**12**_**]**^**3−**^. Based on both spectra, it can be concluded that **[PW**_**11**_**]**^**7−**^ prevails in aqueous solution at pH 6.8. The ^31^P spectrum of the Ni-substituted POT **[PW**_**11**_**Ni]**^**5−**^ (Fig. S8) shows signals at −10.5 and at 482.6 ppm referring to a phosphorus atom within the environment of the Keggin structure, in general agreement with those previously described for **[PW**_**11**_**Ni]**^**5−**^. Assignment is complicated by the special pseudo-paramagnetic character of this cluster (see ^183^W NMR analysis in Fig. S15, SI section 2.2.2.4).

### ^9^Be NMR

In the **[BeW**_**12**_**]**^**6−**^ preparation, the presence of both the α- and β-isomer was detected by ^9^Be NMR (Fig. S9). The reduced symmetry in the chemical environment of the Be center from T_d_ in the α-form to C_3v_ in the β-form results in distinguishable chemical shifts with arbitrary assignment (with no reference for the **[BeW**_**12**_**]**^**6**−^β-isomer available).

### ^183^W NMR

In order to identify the tungstate species responsible for the inhibitory effect observed with the orthotungstate solution **[WO**_**4**_**]**^**2−**^, ^183^W NMR was performed in reaction buffer pH 6.8, which had to be readjusted by a few drops of 5 M HCl to compensate for the marked basicity of **[WO**_**4**_**]**^**2−**^. The ^183^W NMR spectrum (Fig. S10) reveals the complete polycondensation of monomeric orthotungstate to the heptatungstate anion **[W**_**7**_**O**_**24**_**]**^**6−**^, which is confirmed by the presence of three signals. The chemical shifts are in agreement with literature data^56^. Interestingly, albeit comprising a totally different structural archetype and negative charge with respect to the inhibitory Keggin cluster **[SiW**_**12**_**]**^**4−**^, heptatungstate exhibited measurable inhibition effects against *ab*PPO4 activity. The ^183^W NMR spectra for the POT clusters with higher negative charges (see Figs S11–S14) show only one single signal, thereby confirming full structural integrity of the corresponding Keggin anions at pH 6.8. The lacunary cluster **[AlW**_**11**_**]**^**9−**^ evoked a rise in pH up to 8.5 upon dissolution in reaction buffer (pH 6.8) to reach a final concentration of 20 mM. Surprisingly, reacidification to pH 6.8 led to a cluster rearrangement to the monosubstituted anion [AlW_11_O_39_{Al(H_2_O)}]^6−^, as also confirmed by ^27^Al NMR (Fig. S6). In the **[BeW**_**12**_**]**^**6−**^ spectrum (Fig. S14), three additional signals at −147.6, −155.3 and −168.6 ppm can be seen, which were assigned to the β-Keggin isomer. The C_3v_ symmetry of this isomer results in three shifts with intensity ratio 1:2:1, as previously observed for other β-isomers of Keggin structure^60^. The β-isomer is commonly formed during any synthesis of Keggin clusters, but rearranged to the α-form during prolonged thermal reaction. Thus, the occurrence of the β-form in the present preparation can be ascribed to the reduced reaction time applied for the hydrothermal synthesis. Still, as the charge analogue **[H**_**2**_**W**_**12**_**]**^**6−**^ showed exactly the same results as **[BeW**_**12**_**]**^**6−**^ in the presented inhibition studies, the isomeric composition of the POT appeared to be negligible in this case. The ^31^P (cf. Fig. S8) and ^183^W NMR (Fig. S15) spectra of **[PW**_**11**_**Ni]**^**5−**^ have a special character originating from the peculiar electronic configuration of Ni^2+^ and were in general accordance with reference data^61^. In the presented ^183^W NMR spectrum, signals from those W atoms in direct neighbourhood to the Ni^2+^ center cannot be observed, which complicates unambiguous assignment to the desired substituted POT structure. However, the obtained results can be explained in the light of electronic effects. Although the ground state for any distorted octahedral d^8^ complex is diamagnetic (no unpaired electrons), the low excitation energy of many Ni^2+^ complexes to the next electronic state leads to a significant portion of **[PW**_**11**_**Ni]**^**5−**^ being present in a pseudo-paramagnetic state. This results in additional signal splitting by electronic coupling as well as pronounced line-broadening with concomitantly reduced NMR signal intensities, sometimes below the observation limit. Therefore, to confirm the presence of intact **[PW**_**11**_**Ni]**^**5−**^, ESI-MS analysis was also carried out.

### ESI mass spectrometry

The substituted Keggin cluster **[PW**_**11**_**Ni]**^**5−**^ was verified by ESI-MS with an ESI–Qq–oaRTOF (Bruker Daltonics Ltd.) calibrated with an ESI Tuning Mix (Agilent Technologies, USA), and data was evaluated by the corresponding Bruker Daltonics Data Analysis software. Spectra were taken in the *m/z* range from 50 to 1900 with a resolution <5 ppm, covering low *m/z* polytungstate anions (resulting from cluster decomposition in the harsh ESI ionisation conditions) and the specific signals for the intact Keggin cluster as adducts with various cations present in the solution. Negative-ion mode with a capillary voltage of 4500 V was applied for the measurements, and they were conducted in a 1:1 (v/v) H_2_O/ACN mixture.

### Dopachrome assay

For the activity plots shown here, the ratios of reaction rates *v* ([*I*] = 0] and *v*_*app*_ ([*I*] = 0.2–14 mM) were determined from UV absorption measurements at 475 nm using the dopachrome assay. Each reaction setup contained a fixed amount of enzyme (1 µg *ab*PPO4) in 1 mL in a plastic cuvette and the reaction buffer was 50 mM Na-citrate pH 6.8. POT stock solutions were prepared in 20 mM concentration in reaction buffer and adjusted for pH 6.8 with NaOH, if necessary. The reaction rates were extracted from the obtained absorption curves as the slope of linear fits of the steepest curve sections close to the start of the reaction over a time-range of 30 s and were determined in triplicates. The relative enzymatic activity dependent on the inhibitor concentration was fitted to a suitable hyperbolic expression (see SI for details). For determination of inhibition modes, concentration-dependent activity measurements were performed at 5 different *L*-DOPA concentrations ([*S*] = 0.4, 0.6, 0.8, 1.0 and 1.5 mM) for three different inhibitor concentrations, including [*I*] = 0 for the case of no inhibition, an [*I*] value selected to be close to the *K*_*i*_ (as determined from the activity plots) and an additional, usually higher concentration [*I*]. The dopachrome assay was performed exactly as described in section 3.1.1. This time, the observed reaction velocities *v*_*app*_ (in µmol/min) were calculated from the extracted reaction rates (in 1/s) using the extinction coefficient of dopachrome *ε* = 3700 M^−1^ cm^−1 ^^62^. The so obtained data was plotted according to Lineweaver-Burk, and the slopes and ordinate intercepts of these graphs were used to extract inhibition parameters^63^.

## Supplementary information

Supplementary Information
