# Supplementary material for: Keggin-type polyoxotungstates as mushroom tyrosinase inhibitors - A speciation study
Source: Sci Rep. 2019 Mar 26;9:5183. doi: 10.1038/s41598-019-41261-7 (PMC6435698; doi:10.1038/s41598-019-41261-7)
Supplement: Supplementary file 1 — Supplementary Information [file 41598_2019_41261_MOESM1_ESM.pdf]

Supplementary information

Keggin-type polyoxotungstates as mushroom tyrosinase inhibitors - A speciation study

Joscha Breibeck[1], Nadiia I. Gumerova[1], Benedikt B. Boesen[1], Mathea Sophia Galanski[2] and Annette Rompel*[1]

Correspondence to: annette.rompel@univie.ac.at

[1] Dr. Joscha Breibeck, Nadiia Gumerova, PhD, Benedikt Boesen, BSc, Annette Rompel, Universität Wien, Fakultät für Chemie, Institut für Biophysikalische Chemie, Althanstraße 14, 1090 Wien, Austria; http://www.bpc.univie.ac.at,

[2] Ao. Univ.-Prof. Dr. Mathea Sophia Galanski, Universität Wien, Fakultät für Chemie, Institut für Anorganische Chemie, Währinger Straße 42, 1090 Wien, Austria

Contents

1. Preparation of *ab*PPO4 in its active form....3
2. Preparation of Keggin POTs....4
   2.1 Structural isomers of the Keggin archetype....4
   2.2 Characterization of α-Keggin POTs....6
      2.2.1 IR spectroscopy....9
      2.2.2 NMR spectroscopy....11
         2.2.2.1 ^{27}Al NMR....11
         2.2.2.2 ^{31}P NMR....12
         2.2.2.3 ^{9}Be NMR....13
         2.2.2.4 ^{183}W NMR....14
      2.2.3 ESI mass spectrometry....18
3. Enzyme kinetics....19
   3.1 Kinetic evaluation according to general mixed inhibition model....19
   3.2 Determination of inhibition type....20
      3.2.1 Kinetic evaluation using Lineweaver-Burk plots....20
      3.2.2 Kinetic evaluation using Dixon plots....21
4. Abbreviations....32
5. References....32

1. Preparation of *ab*PPO4 in its active form

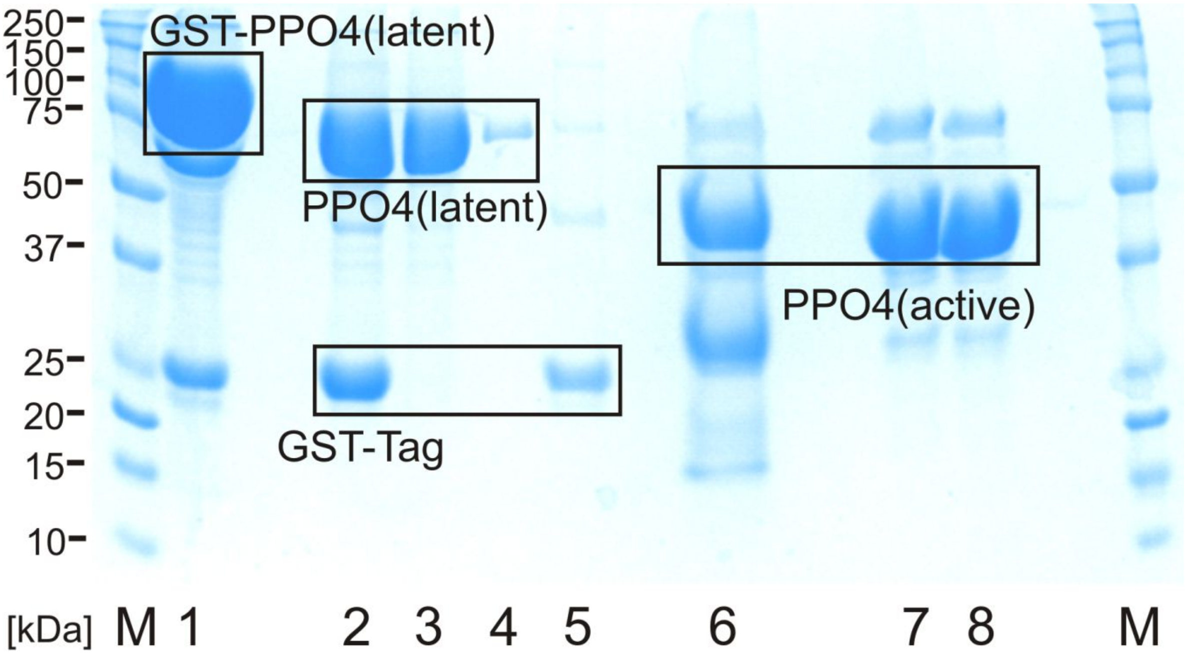

Figure S1. SDS-PAGE of the chromatographic purification of recombinant *ab*PPO4 in its active form according to Pretzler *et al.*[1]. For preparation of this figure, the gel was slightly cropped at the edges (see Figure S24 for the original image). Lane M: Precision Plus Protein™ Standard Dual Color (Bio-Rad Laboratories), 1: GST-PPO4(latent) fusion protein after first GST chromatography, 2: GST-PPO4(latent) cleavage reaction with HRV 3C protease, 3 and 4: flowthrough with free PPO4(latent) after second GST chromatography, 5: eluate of second GST chromatography, 6: PPO4(latent) cleavage reaction with Proteinase K, 7 and 8: PPO4(active) after SEC chromatography. Calculated molecular weights: GST-PPO4(latent), 91.5 kDa; PPO4(latent), 65.1 kDa; PPO4(active), 44.5 kDa; GST-Tag, 26.4 kDa.

2. Preparation of Keggin POTs

2.1 Structural isomers of the Keggin archetype

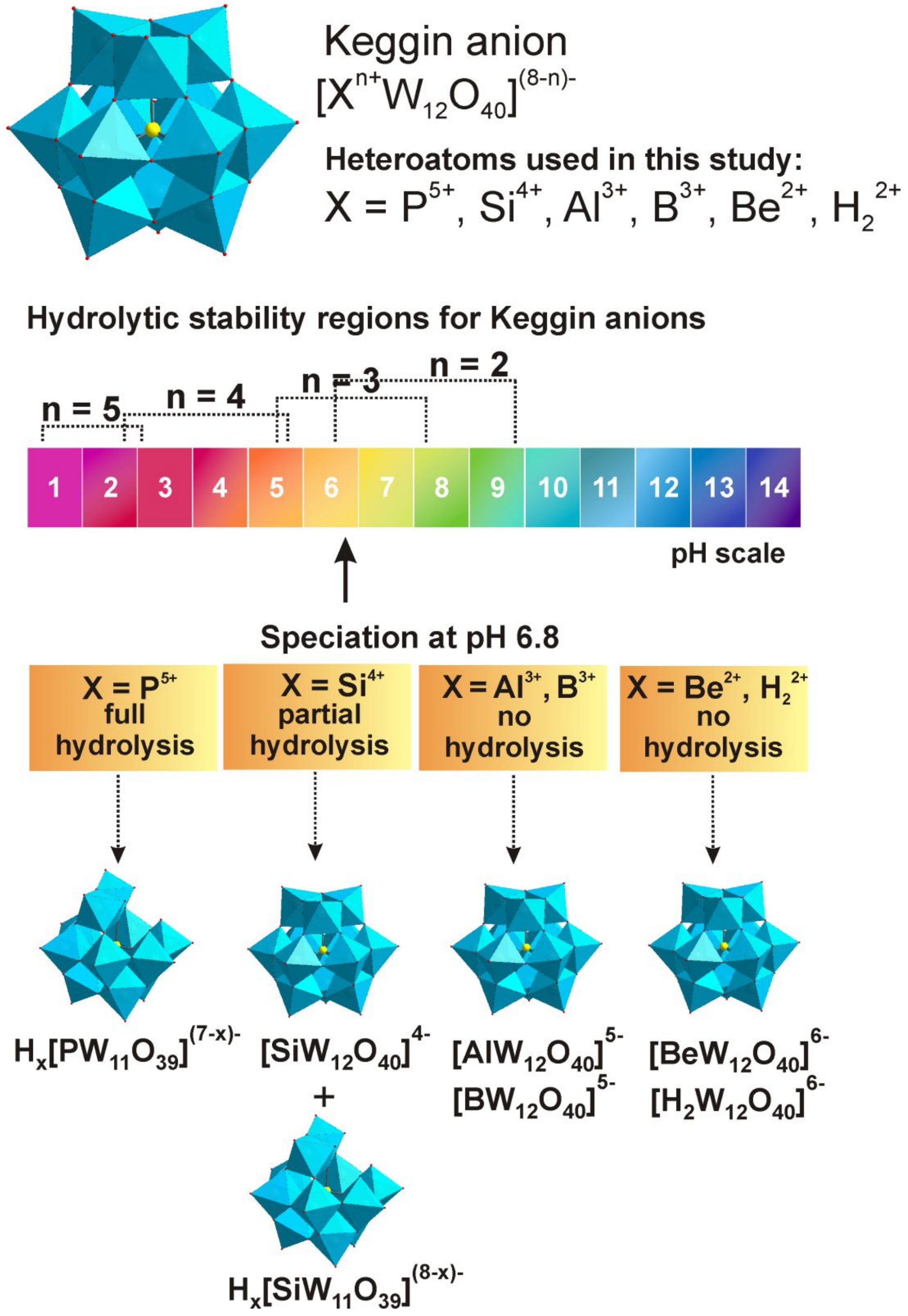

Figure S2. Summary of the NMR speciation of non-substituted Keggin POT anions performed in this study. The higher the negative net charge of the POT anion, the more stable against hydrolysis it appears to be at the given pH 6.8. The Keggin clusters with lower charge were proven to be affected by pronounced hydrolysis. While **$[SiW_{12}]^{4-}$** was only partially hydrolyzed to its lacunary form, **$[PW_{12}]^{3-}$** was completely converted, and only the POTs with charges 5- and 6- retained full structural integrity. Color code: $\{WO_6\}$, blue octahedra; central heteroatom, yellow sphere; O, small red spheres. Metatungstate **$[H_2W_{12}]^{6-}$** comprises two protons in its central tetrahedral cavity.

The pH-dependent speciation of the non-substituted Keggin POTs under investigation according to the extensive NMR analyses shown in section 2.2.2 below is presented in Figure S2. The oxidation state of the incorporated heteroatom determines the negative net charge of the POT anion. With increasing negative charge, hydrolytic decomposition is prevented more efficiently.

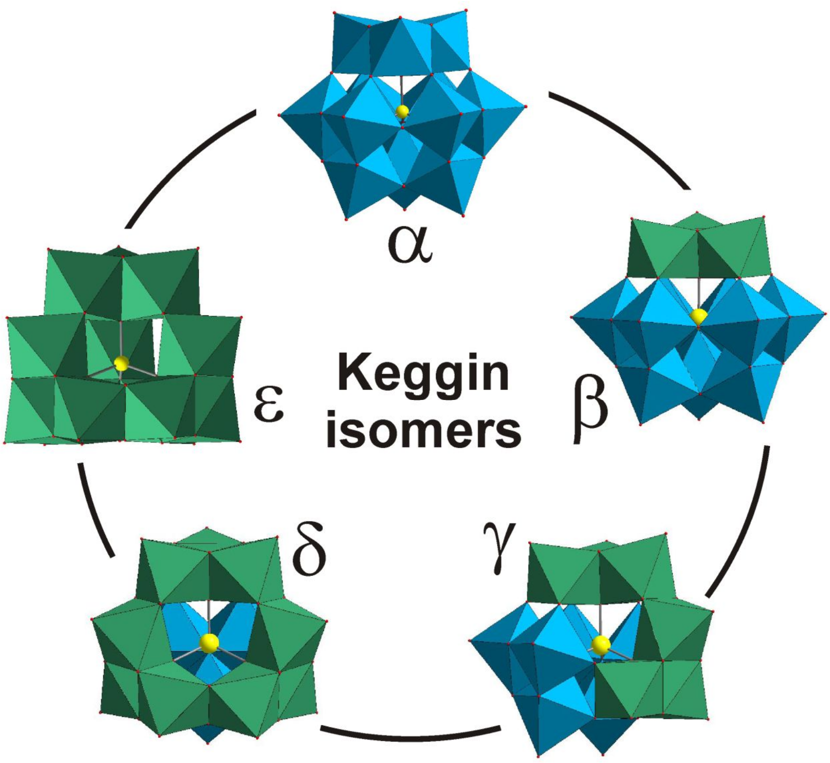

Figure S3. Five structural isomers of the Keggin archetype of polyoxometalates. The α-isomer is the energetically most favoured and synthetically best accessible isomer and was therefore used as a common scaffold for all the POTs in this study. The other isomers can be obtained by stepwise rotation of the four $\{M_3O_{13}\}$ triads. Color code: not rotated $\{WO_6\}$, blue; rotated $\{WO_6\}$, green; central heteroatom, yellow; O, red.

In accordance with the tetrahedral symmetry of the α-Keggin polyoxometalate archetype, the cluster can be grouped into four $\{M_3O_{13}\}$ triads. Rotation of one triad about 60° results in another isomeric structure. The γ-, δ- and ε-Keggin isomers are usually reported only for mixed addenda atom compositions or for structures stabilized by covalent modification with organic moieties or 3d- and 4-f metals. There are synthetic procedures for the preparation of β-isomers, but only for a limited selection of different heteroatoms. The α-Keggin isomer is the most stable form for its highly symmetrical architecture and is accessible for a large array of heteroatoms, which is why it served the basic POT structure throughout this study[2].

2.2 Characterization of α-Keggin POTs

Previous to the enzymatic inhibition studies, the Keggin POTs were verified by IR spectroscopy (see section 2.2.1). In the course of NMR speciation (see section 2.2.2), all the POT anions were confirmed to correspond to the α-isomer. Only the **$[BeW_{12}]^{6-}$** preparation was revealed as a mixture of the α- and β-isomer, however, leaving the experimental consistency and data interpretation unaffected due to direct comparability with **$[H_2W_{12}]^{6-}$**. As neither ^{183}W nor ^{31}P NMR provided unambiguous evidence for the substituted POT **$[PW_{11}Ni]^{5-}$**, additional ESI-MS was performed to directly prove the intact cluster.

Table S1. Keggin POT clusters synthesized in this study. If not stated otherwise, NMR signals are given as obtained in 50 mM Na-citrate pH 6.8.

Keggin POT	Abbreviation	^{183}W NMR (δ_W) [ppm]	^{31}P (δ_P), ^{27}Al (δ_{Al}) and ^{9}Be (δ_{Be}) NMR [ppm]	IR [cm^{-1}]	Reference		
					Synthesis according to	First structural report by	NMR assignment according to
$Na_3[PW_{12}O_{40}]$ x 9 H_2O	**$[PW_{12}]^{3-}$**	–94.1, –97.8, –103.0, –110.0, –117.4, –132.9, –153.4	+4.5, +3.8, +2.5, –2.2, –3.6, –5.7, –7.1, –7.4, –9.2, –10.8, –11.4, –14.4 (δ_P)	769, 511, 960, 888	[3]	[4]	[5],[6],[7]
$K_4[SiW_{12}O_{40}]$ x 14 H_2O	**$[SiW_{12}]^{4-}$**	–99.4, –101.9, –115.0, –120.6, –128.0, –141.5, –175.3		748, 919, 521, 467, 881, 970	[8]	[9]	[10]
$K_5[BW_{12}O_{40}]$ x 18 H_2O	**$[BW_{12}]^{5-}$**	–128.8		785, 741, 500, 470, 898, 425, 954	[11]	[12]	[13]
$Na_5[AlW_{12}O_{40}]$ x 15 H_2O	**$[AlW_{12}]^{5-}$**	–109.6	–72.5 (δ_{Al})	746, 468, 795, 530, 878, 953	[14]	[15]	[13],[14]
$Na_6[H_2W_{12}O_{40}]$ x 18 H_2O	**$[H_2W_{12}]^{6-}$**	–117.5		710, 864, 930, 405, 951	[16]	[17]	[13]
$Na_6[BeW_{12}O_{40}]$ x 6 H_2O	**$[BeW_{12}]^{6-}$**	–144.5, –147.6, –155.3, –168.6	–0.16, –0.21 (δ_{Be})	774, 412, 876, 931, 532, 576, 467, 974	[18]		
$K_5[PW_{11}O_{39}\{Ni(H_2O)\}]$ x 11 H_2O	**$[PW_{11}Ni]^{5-}$**	+617.8, –68.4, –119.1, –197.7	+482.6, +1.4, –10.5 (δ_P)	797, 694, 759, 960, 506, 484, 410, 879	[19]	[20]	[21]
Heptatungstate (not Keggin archetype)	**$H[W_7O_{24}]^{5-}$**	+268.8, –90.9, –180.2					[22],[23]

Keggin POT	Abbreviation	^{183}W NMR (δ_W) [ppm]	^{31}P (δ_P), ^{27}Al (δ_{Al}) and ^{9}Be (δ_{Be}) NMR [ppm]	IR [cm^{-1}]	Reference		
					Synthesis according to	First structural report by	NMR assignment according to
$K_7[PW_{11}O_{39}]$ x 9 H_2O	**$[PW_{11}]^{7-}$**	–96.1, –101.9, –107.9, –116.0, –131.1, –152.3	–10.9 (δ_P)	945, 902, 856, 798. 715, 509, 503, 430	[24]	[25]	[5],[6],[7]
$K_8[SiW_{11}O_{39}]$ x 6 H_2O	**$[SiW_{11}]^{8-}$**	–98.1, –114.4, –120.5, –127.5, –141.0, –175.2		954, 864, 790, 709, 534, 505, 467	[8]	[26]	[10]
$Na_9[AlW_{11}O_{39}]$ x 16 H_2O *	**$[AlW_{11}]^{9-}$**	–97.6, –121.9, –139.2, –147.6, –173.7, –199.7	–67.0 (δ_{Al})	923, 835, 750, 684, 456	[15]	[15]	[15]

* taken at pH 8.5

2.2.1 IR spectroscopy

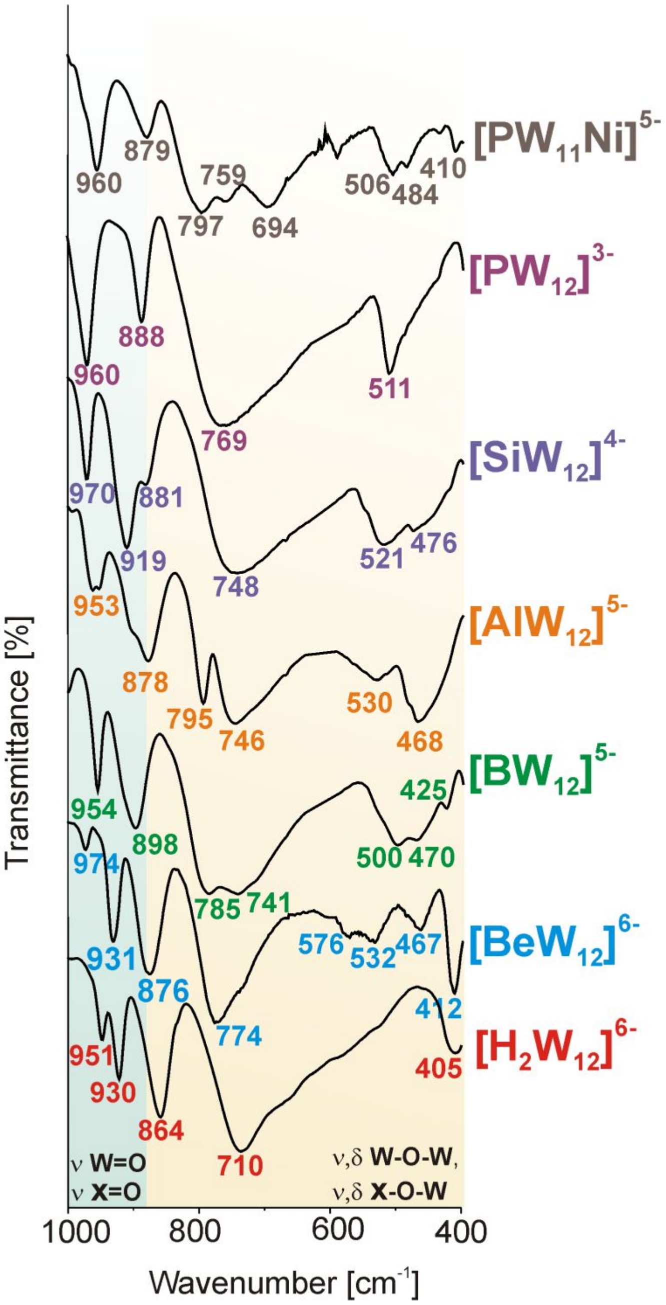

Figure S4. IR spectra of **$[XW_{12}]^{n-}$** (X = H_2^{2+}, Be^{2+}, B^{3+}, Al^{3+}, Si^{4+}, P^{5+}) and **$[PW_{11}Ni]^{5-}$** in the region of W–O–W and W=O bonds vibration (1000 – 400 cm^{-1}).

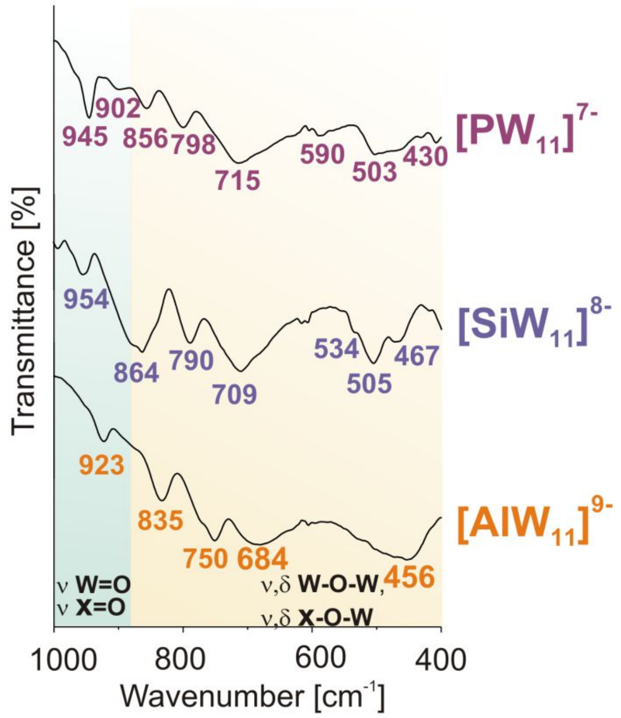

Figure S5. IR spectra of **$[XW_{11}]^{n-}$** (X = Al^{3+}, Si^{4+}, P^{5+}) in the region of W–O–W and W=O bonds vibration (1000 – 400 cm^{-1}).

2.2.2 NMR spectroscopy

2.2.2.1 ^{27}Al NMR

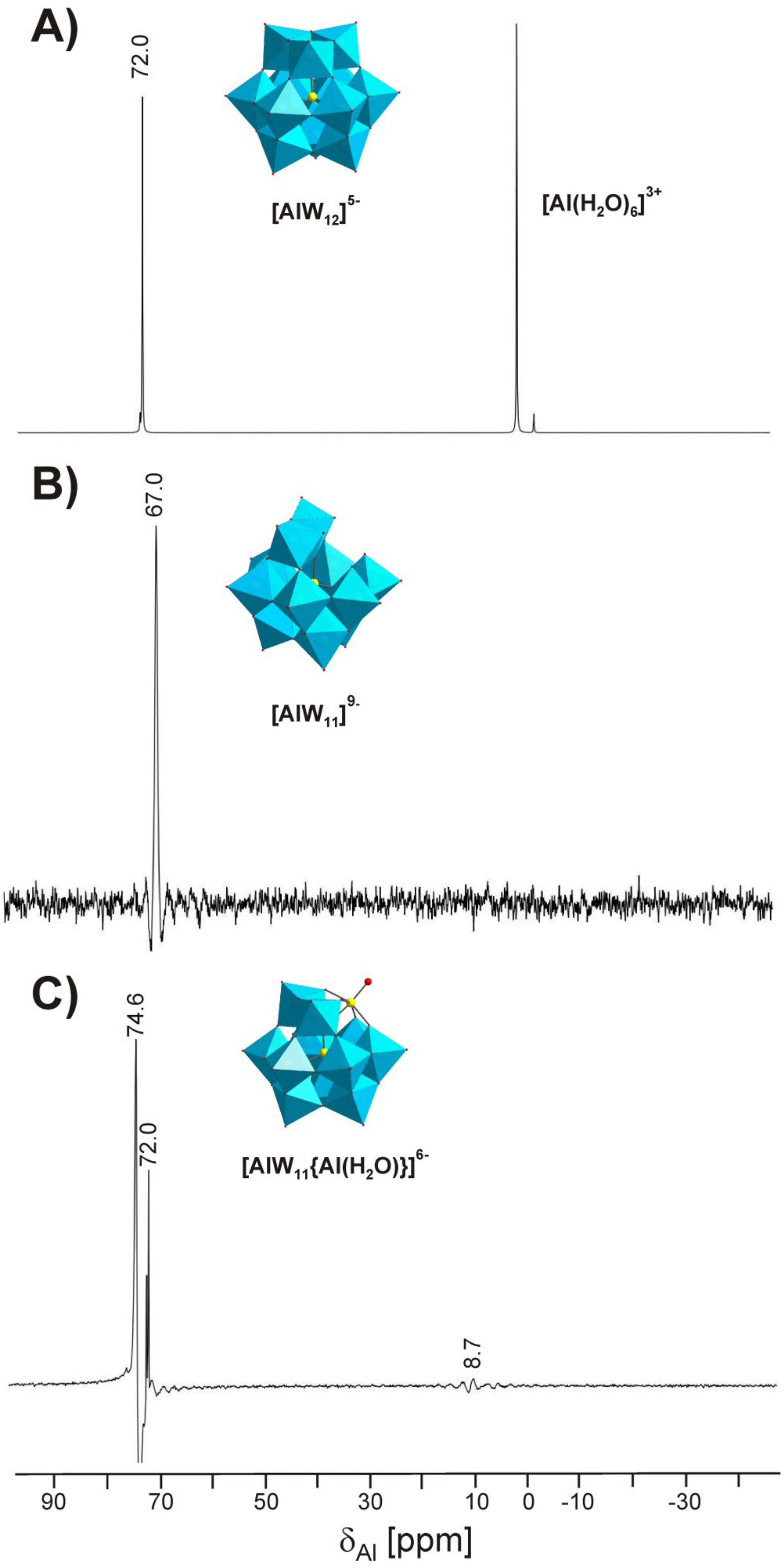

Figure S6. ^{27}Al NMR spectra of Al-containing POTs with 10% D_2O. A) Reaction mixture during $\mathbf{[AlW_{12}]^{5-}}$ synthesis, B) lacunary anion $\mathbf{[AlW_{11}]^{9-}}$ in solution pH 8.5, C) $\mathbf{[AlW_{11}]^{9-}}$ after pH adjustment to 6.8. Acidification of the $\mathbf{[AlW_{11}]^{9-}}$ solution resulted in formation of $[AlW_{11}\{Al(H_2O)\}]$. Color code: $\{WO_6\}$, blue; Al, yellow; O, red.

2.2.2.2 ^{31}P NMR

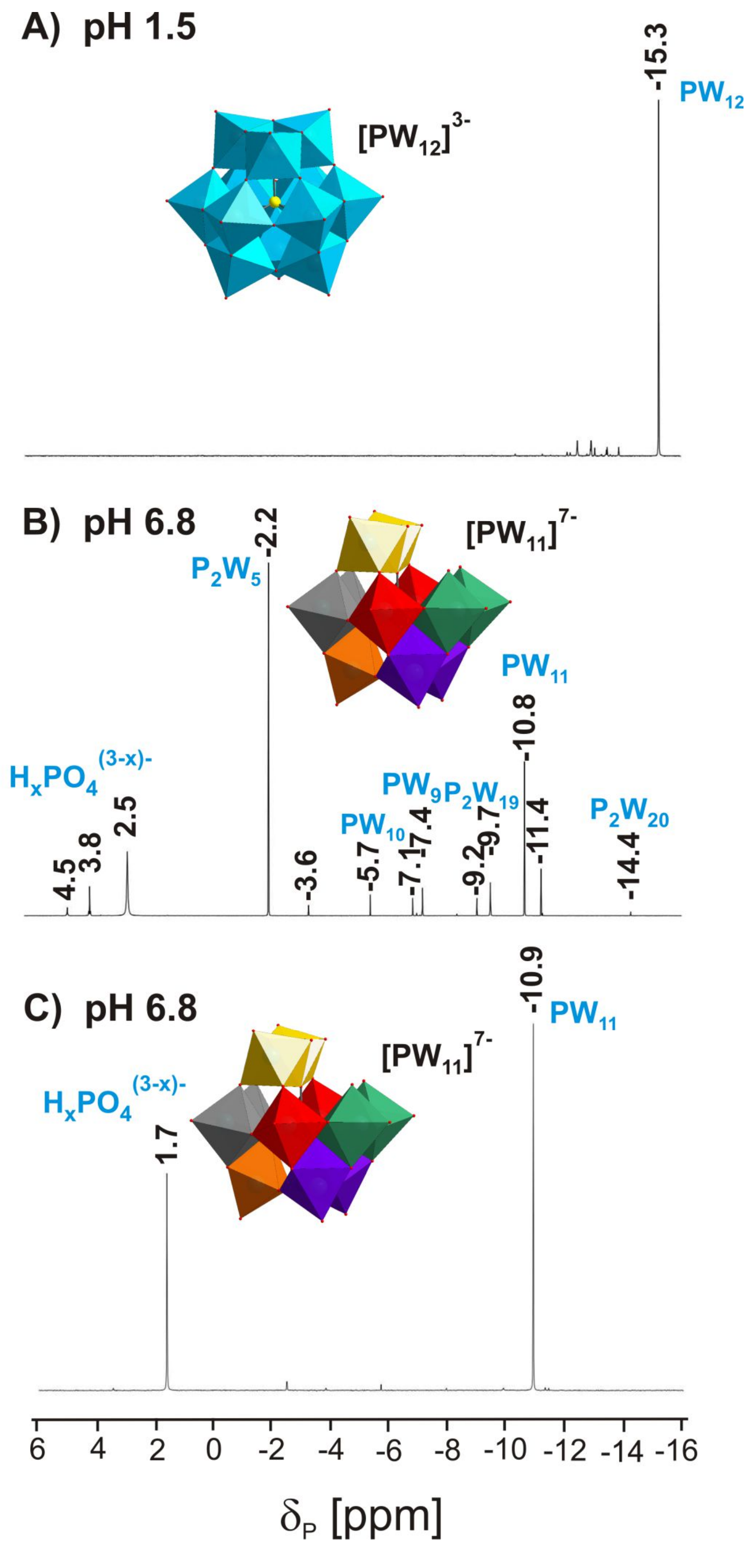

Figure S7. ^{31}P NMR spectra of **[PW$_{12}$]$^{3-}$** A) at pH 1.5 and B) at pH 6.8 with 10% D_2O. No intact Keggin anion **[PW$_{12}$]$^{3-}$** was detectable at pH 6.8 (cf. Figure 4D). The lacunary anion **[PW$_{11}$]$^{7-}$** is depicted in colours representing the symmetry-equivalent W positions in order to illustrate the less symmetric environment experienced by the P center. C) ^{31}P NMR spectrum of **[PW$_{11}$]$^{7-}$** (synthesized from **[PW$_{12}$]$^{3-}$**) at pH 6.8. The lacunary anion remains the only P-containing POT species in solution. Color code: {WO_6}, blue, green, red, grey, yellow, orange, dark-blue; P, yellow; O, red.

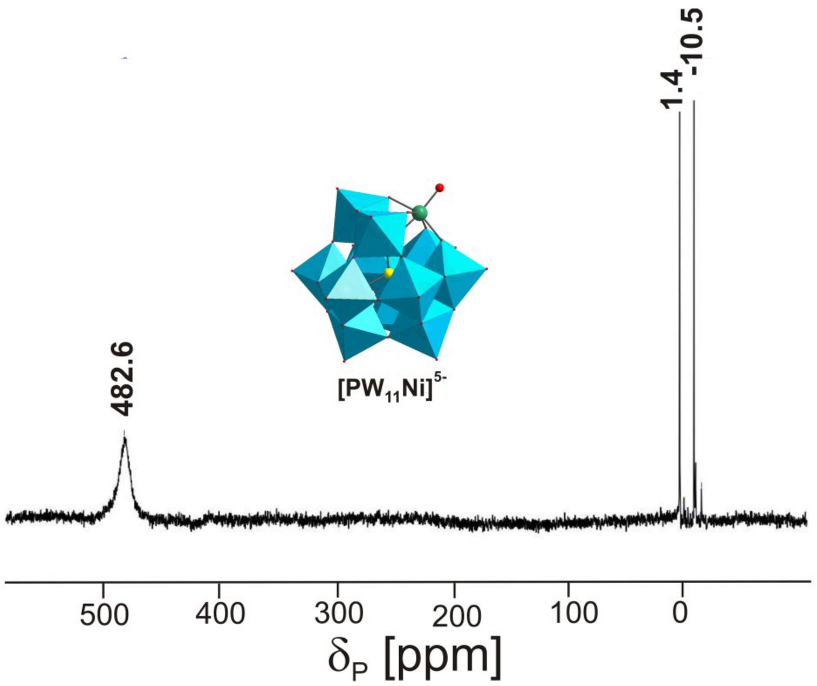

Figure S8. ^{31}P-NMR spectrum of **$[PW_{11}Ni]^{5-}$** at pH 6.8 with 10% D_2O. Color code: {WO_6}, blue; P, yellow; Ni, green; O, red.

2.2.2.3 ^{9}Be NMR

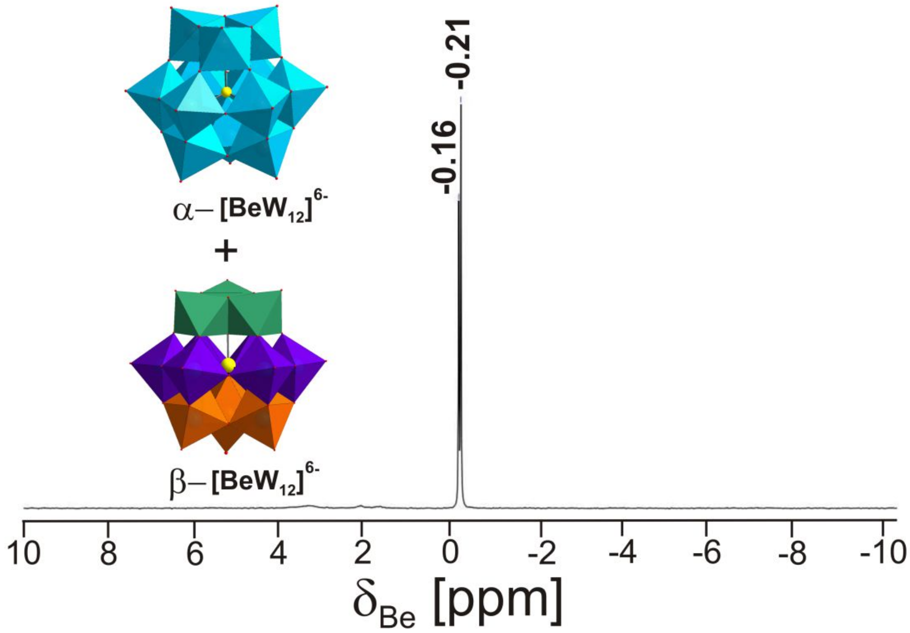

Figure S9. ^{9}Be NMR spectrum of **$[BeW_{12}]^{6-}$** at pH 6.8 with 10% D_2O. The spectrum features two distinguishable signals, in accordance with the two Keggin isoforms determined by ^{183}W NMR (cf. Figure S14). Signal assignment is arbitrary due to the unprecedented β-isomer and the close proximity of the signals. The β-isomer is depicted in colours representing the symmetry-equivalent W positions in order to illustrate the less symmetric environment experienced by the Be center. Color code: {WO_6}, blue, green, dark-blue, orange; Be, yellow; O, red.

2.2.2.4 ^{183}W NMR

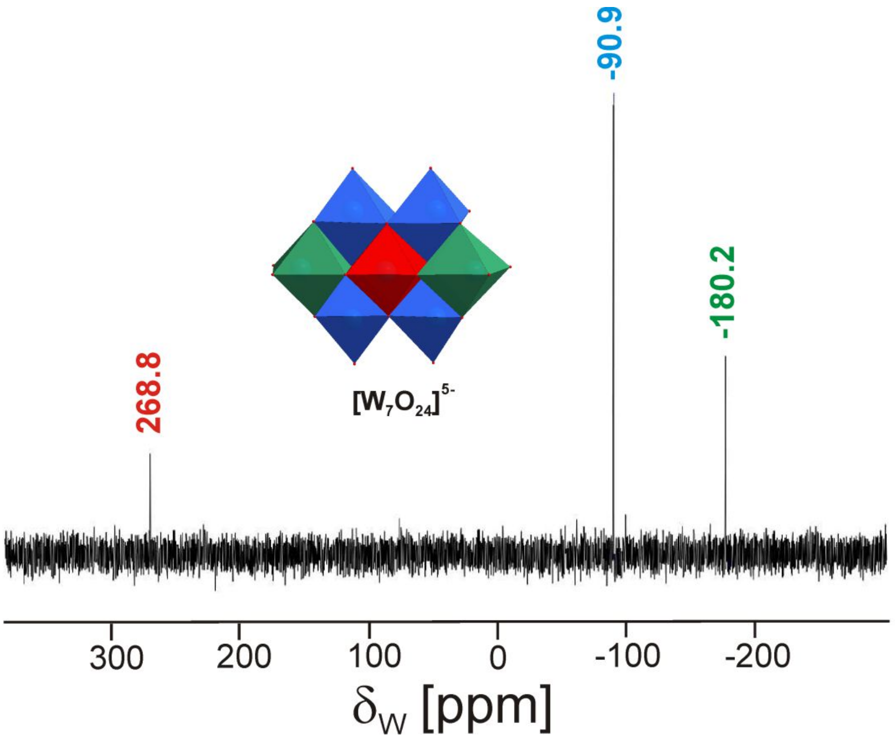

Figure S10. ^{183}W NMR spectrum of **$[WO_4]^{2-}$** solution at pH 6.8 with 10% D_2O. The NMR signals are assigned to W nuclei sharing the same chemical environment according to literature data[23]. The total recording time is about 60 hours for the measurement, chemical shifts were measured relative to external 1 M Na_2WO_4. Color code: {WO_6}, blue, green, red; O, red.

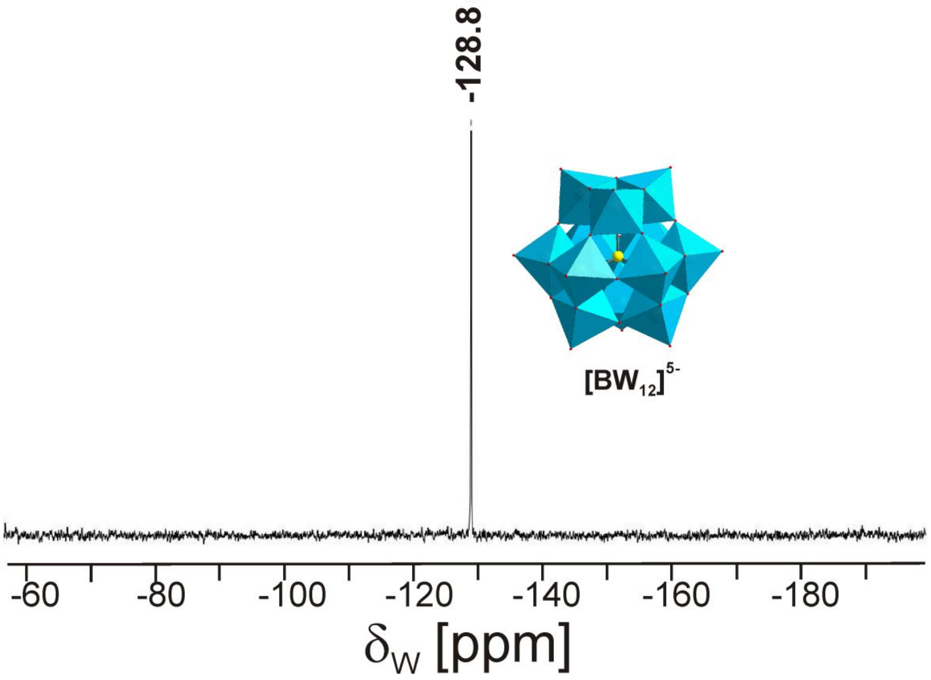

Figure S11. ^{183}W-NMR spectrum of **$[BW_{12}]^{5-}$** at pH 6.8 with 10% D_2O. The total recording time is about 60 hours for the measurement, chemical shifts were measured relative to external 1 M Na_2WO_4. Color code: {WO_6}, blue; B, yellow; O, red.

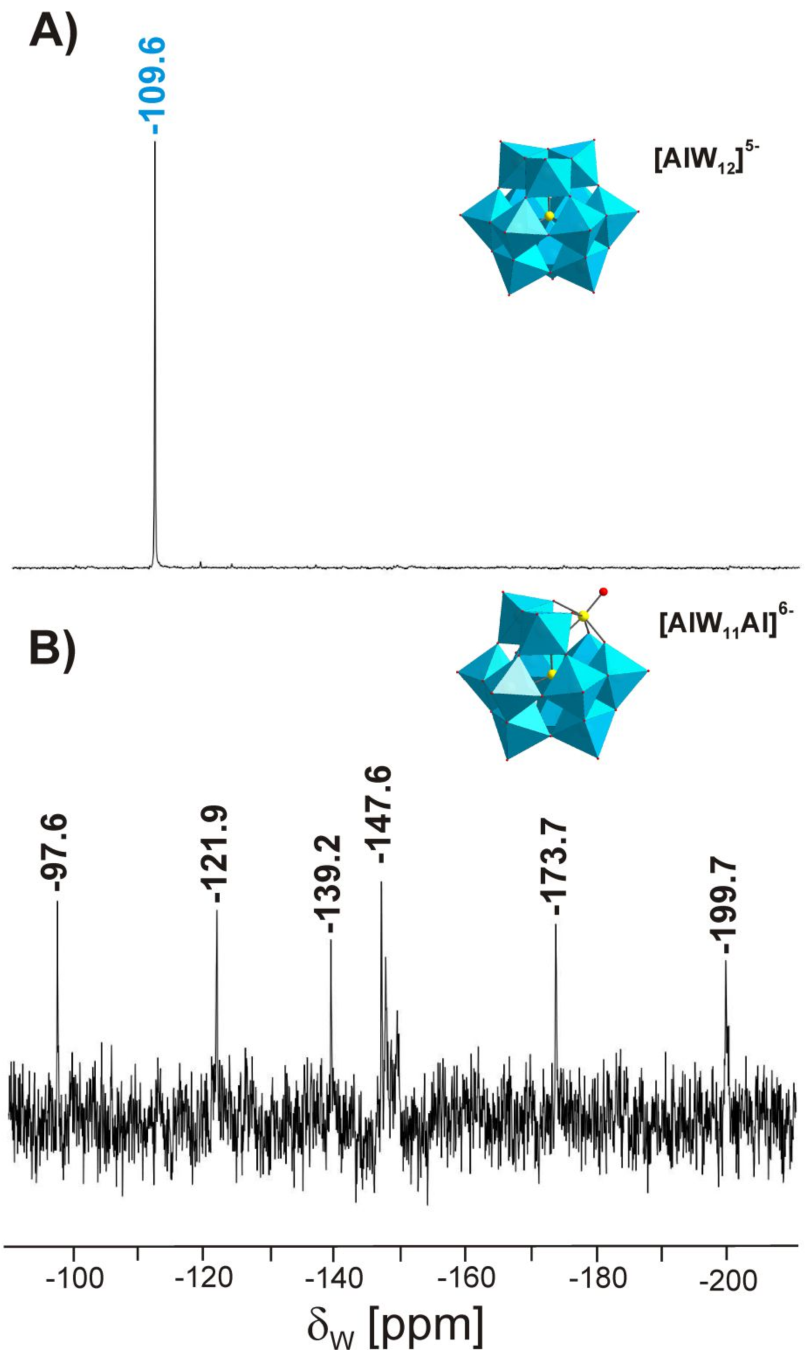

Figure S12. ^{183}W NMR spectra of Al-Keggin POTs A) $\mathbf{[AlW_{12}]^{5-}}$ and B) $\mathbf{[AlW_{11}]^{9-}}$ at pH 6.8 with 10% D_2O. A) $\mathbf{[AlW_{12}]^{5-}}$ is characterized by a single peak due to its high symmetry. B) At pH 6.8, the lacunary anion $\mathbf{[AlW_{11}]^{9-}}$ rearranges to the monosubstituted cluster $[AlW_{11}\{Al(H_2O)\}]^{6-}$, which explains the unexpected inhibitory capacity observed for this compound. The total recording time is about 60 hours for the measurement, chemical shifts were measured relative to external 1 M Na_2WO_4. Color code: $\{WO_6\}$, blue; Al, yellow; O, red.

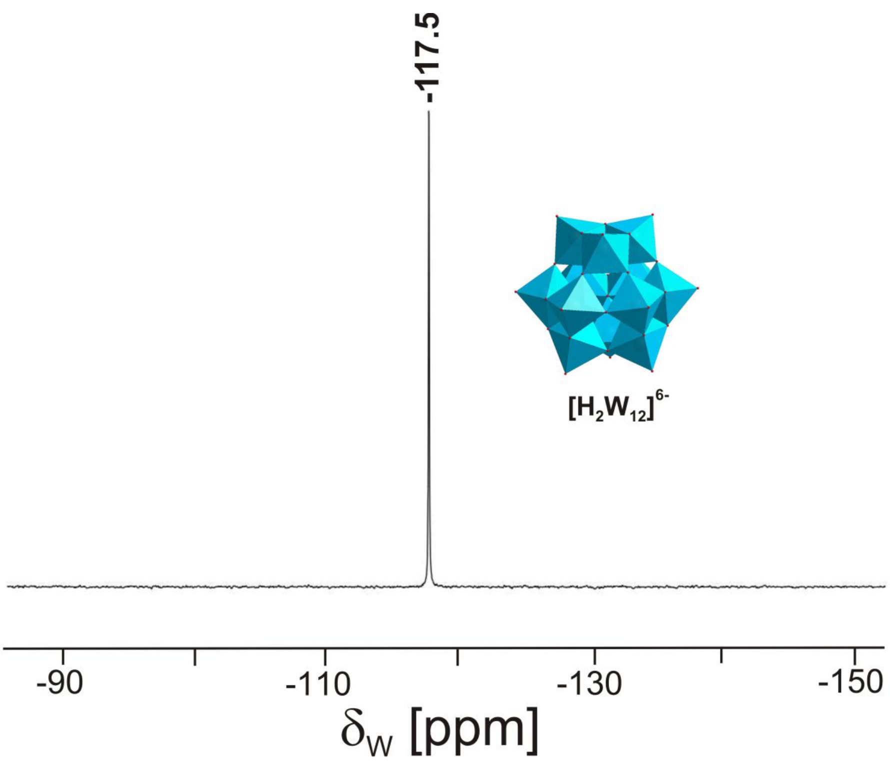

Figure S13. ^{183}W NMR spectrum of $\mathbf{[H_2W_{12}]^{6-}}$ at pH 6.8 with 10% D_2O. The total recording time is about 60 hours for the measurement, chemical shifts were measured relative to external 1 M Na_2WO_4. Color code: $\{WO_6\}$, blue; O, red. Metatungstate comprises two protons in its central tetrahedral cavity.

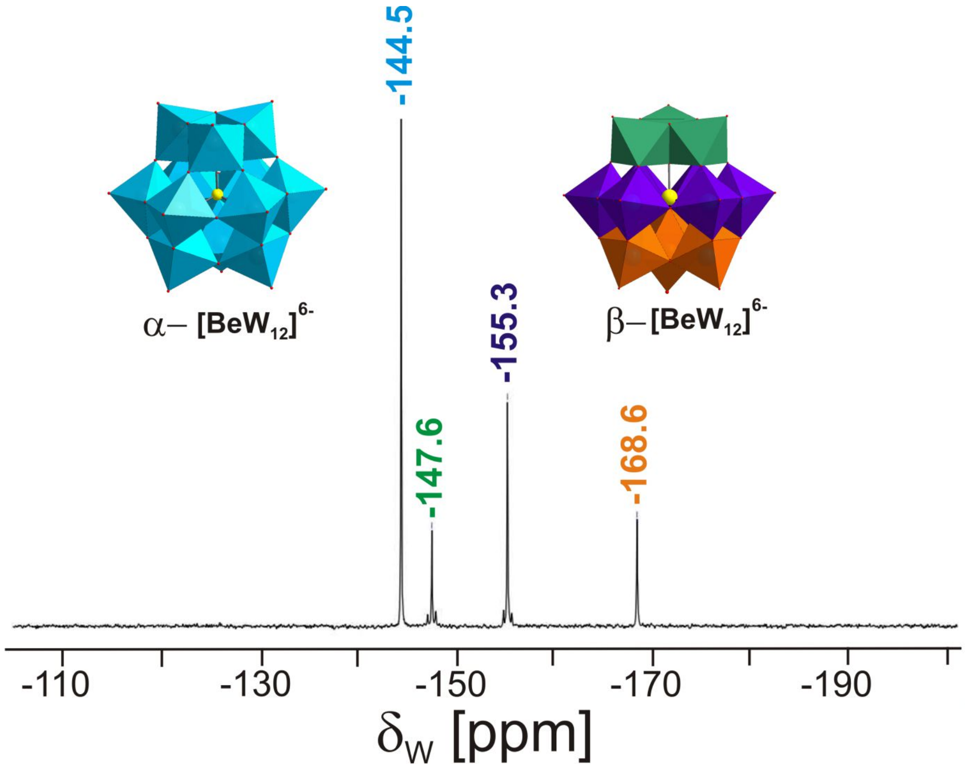

Figure S14. ^{183}W NMR spectrum of $\mathbf{[BeW_{12}]^{6-}}$ at pH 6.8 with 10% D_2O. The total recording time is about 60 hours for the measurement, chemical shifts were measured relative to external 1 M Na_2WO_4. Color code: $\{WO_6\}$, blue, green, dark-blue, orange; Be, yellow; O, red. The ^{183}W NMR signals are assigned to W nuclei sharing the same chemical environment according to literature data[27] and can be assigned to the α- and β-Keggin isomer.

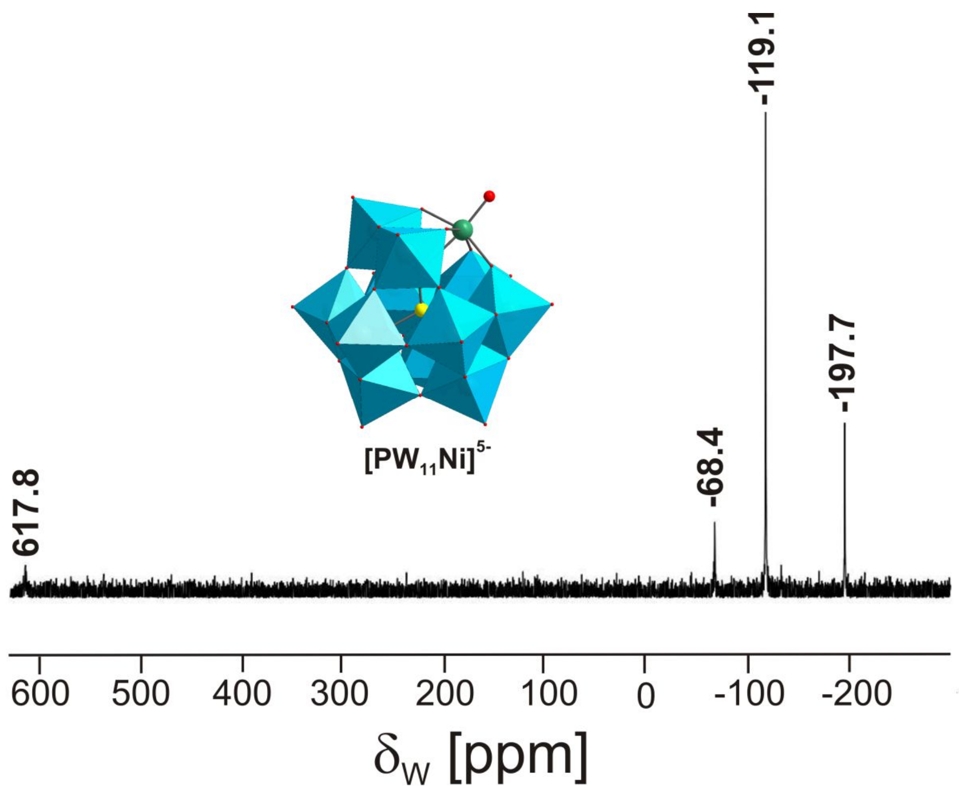

Figure S15. ^{183}W NMR spectrum of $\mathbf{[PW_{11}Ni]^{5-}}$ at pH 6.8 with 10% D_2O. The total recording time is about 60 hours for the measurement, chemical shifts were measured relative to external 1 M Na_2WO_4. Color code: $\{WO_6\}$, blue; P, yellow; Ni, green; O, red.

2.2.3 ESI mass spectrometry

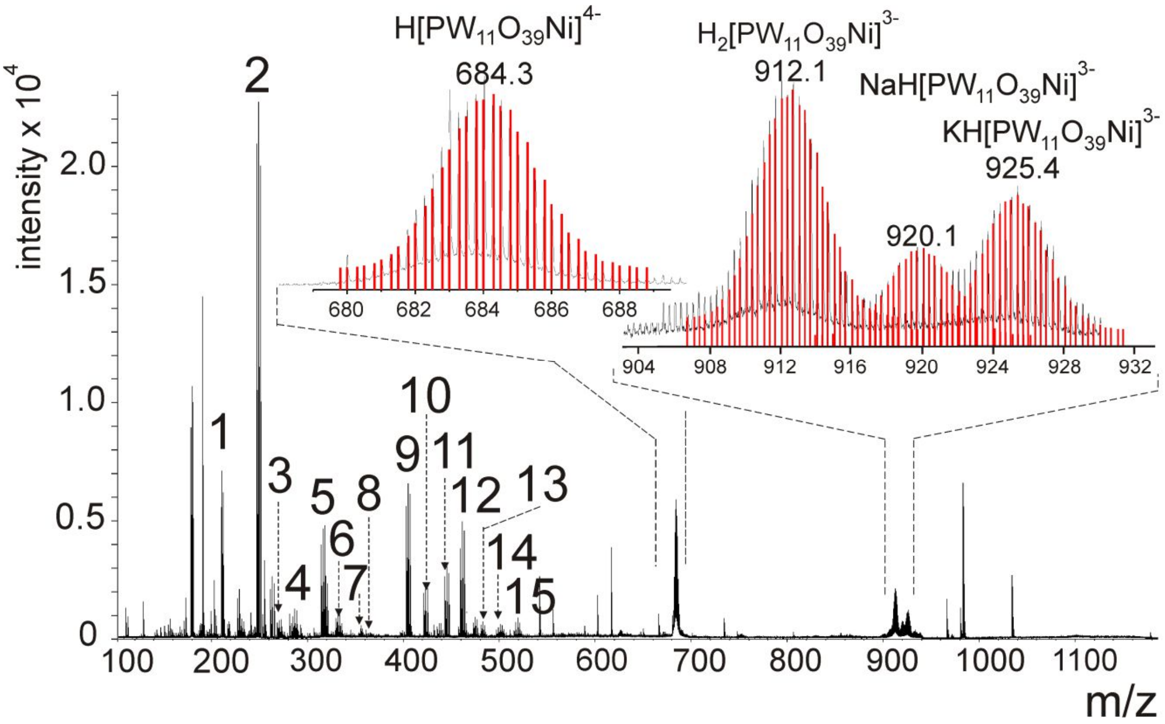

Figure S16. ESI-MS spectrum of $\mathbf{[PW_{11}Ni]^{5-}}$ (in H_2O/ACN) in negative mode. Interestingly, the loose water ligand attached to the Ni^{2+} center in solid state and aqueous solution apparently went off under the electrospray ionisation conditions.

Table S2. Assignment of ESI-MS signals for $\mathbf{[PW_{11}Ni]^{5-}}$ and its derivatives upon ESI fragmentation (cf. Figure S16).

**Signal number**	***m/z***	***m/z (calc.)***	**Peak assignment to fragment**
1	209.9	210.0	$H_3[PWNiO_9]^{2-}$
2	248.9	248.9	$H[WO_4]^-$
3	270.9	270.9	$Na[WO_4]^-$
4	286.9	286.9	$K[WO_4]^-$
5	316.8	316.8	$Na_2H_2[NiW_2O_{10}]^{2-}$
6	332.9	333.0	$K_2H_2[NiW_2O_{10}]^{2-}$
7	346.9	346.9	$KNa_3[NiW_2O_{10}]^{2-}$
8	355.9	355.9	$[W_3O_{10}]^{2-}$
9	402.9	402.9	$Na_2H[WNiO_6(H_2O)]^-$
10	420.9	421.0	$H_4[PWNiO_9]^-$
11	442.8	442.9	$NaH_3[PWNiO_9]^-$
12	458.8	458.9	$KH_3[PWNiO_9]^-$
13	480.9	480.9	$H[W_2O_7]^-$
14	502.9	502.9	$Na[W_2O_7]^-$
15	518.9	518.9	$K[W_2O_7]^-$

3. Enzyme kinetics

(The kinetic models with according mathematical expressions applied in this study are based on Copeland's guide for enzyme kinetics[28].)

3.1 Kinetic evaluation according to general mixed inhibition model

The classical Michaelis-Menten equation[28] relates the velocity *v* of an enzymatic reaction to the applied substrate concentration *[S]*:

$$v = v_{max} \cdot \frac{[S]}{K_M + [S]} \qquad (1)$$

In this equation, the Michaelis constant K_M represents the substrate concentration effecting the half-maximum reaction rate v_{max}.

For the reported inhibition studies, we chose an expanded version of this equation (1) which accounts for all possible inhibition mechanisms and combinations thereof[28]. In general, both v_{max} and K_M can be affected upon enzyme inhibition, resulting in the (usually reduced) altered apparent values:

$$v_{max,app} = \frac{v_{max}}{1 + \frac{[I]}{\alpha \cdot K_i}} \qquad (2)$$

and

$$K_{M,app} = \frac{K_M \cdot \left(1 + \frac{[I]}{K_i}\right)}{1 + \frac{[I]}{\alpha \cdot K_i}} \qquad (3)$$

Herein, *[I]* refers to the inhibitor concentration, K_i is the inhibition constant measuring the inhibitory capacity and the parameter α gives information on the exhibited mode of inhibition. The three ideal inhibition types are reflected by three limiting conditions for α:

(A) $0 < \alpha \ll 1$: uncompetitive inhibition

(B) $\alpha = 1$: non-competitive inhibition

(C) $\alpha \gg 1$: competitive inhibition

Therefore, values of $\alpha < 1$ often correspond to mixed-type inhibition with contributions from uncompetitive and non-competitive inhibition modes.

Insertion of expressions (2) and (3) in equation (1) leads to the final mixed-type inhibition model:

$$v_{app} = v_{max,app} \cdot \frac{[S]}{K_{M,app}+[S]} = \frac{v_{max}}{1+\frac{[I]}{\alpha \cdot K_i}} \cdot \frac{[S]}{\frac{K_M \cdot (1+\frac{[I]}{K_i})}{1+\frac{[I]}{\alpha \cdot K_i}}+[S]} \quad (4)$$

The relative enzymatic activity upon inhibition can be expressed as the ratio of the observed reaction velocity v_{app} to the non-inhibited reaction performance v through dividing equation (4) by equation (1):

$$\frac{v_{app}}{v} = \frac{\frac{v_{max}}{1+\frac{[I]}{\alpha \cdot K_i}} \cdot \frac{[S]}{\frac{K_M \cdot (1+\frac{[I]}{K_i})}{1+\frac{[I]}{\alpha \cdot K_i}}+[S]}}{v_{max} \cdot \frac{[S]}{K_M+[S]}} = \frac{K_M+[S]}{K_M \cdot \left(1+\frac{[I]}{K_i}\right) + [S] \cdot \left(1+\frac{[I]}{\alpha \cdot K_i}\right)} \quad (5)$$

This corresponds to a normalization of the reaction rates with respect to the non-inhibited rate. As we were interested in the dependence of the reaction velocity on the inhibitor concentration, the substrate concentration was fixed to 1 mM *L*-DOPA for all activity plots. This led to a further simplification of equation (1):

$$\frac{v_{app}}{v}[in\ \%] = 100 \cdot \frac{K_M+1}{K_M \cdot \left(1+\frac{[I]}{K_i}\right) + \left(1+\frac{[I]}{\alpha \cdot K_i}\right)} = 100 \cdot \frac{K_M+1}{K_M + 1 + \left(\frac{K_M+\frac{1}{\alpha}}{K_i}\right) \cdot [I]} \quad (6)$$

Equation (6) was used as the final curve fit expression for kinetic evaluation of those POM compounds showing inhibitory activity. K_M was set to 26.1 mM as reported for *ab*PPO4 by Pretzler *et al.*[1]. The fitted α-parameters were smaller than 1, but not close to zero, suggesting a mixed-type inhibition as a combination of uncompetitive and non-competitive contributions.

3.2 Determination of inhibition type

3.2.1 Kinetic evaluation using Lineweaver-Burk plots

The inhibition mode was determined for each compound with inhibitory effects by analysis of linear Lineweaver-Burk[29] extrapolations. The double-reciprocal plots of $1/v_{app}$ against 1/[S] yielded straight lines that were fitted accordingly to the correspondingly rearranged form of equation (4):

$$\frac{1}{v_{app}} = \frac{1}{\frac{v_{max}}{1+\frac{[I]}{\alpha \cdot K_i}} \cdot \frac{[S]}{\frac{K_M \cdot \left(1+\frac{[I]}{K_i}\right)}{1+\frac{[I]}{\alpha \cdot K_i}}+[S]}} = \frac{1}{\frac{v_{max}}{1+\frac{[I]}{\alpha \cdot K_i}} \cdot \frac{[S]}{\frac{K_M \cdot \left(1+\frac{[I]}{K_i}\right)+[S] \cdot \left(1+\frac{[I]}{\alpha \cdot K_i}\right)}{1+\frac{[I]}{\alpha \cdot K_i}}}}$$

$$= \frac{1}{v_{max}} \cdot \left(\frac{K_M \cdot \left(1+\frac{[I]}{K_i}\right)}{[S]} + 1 + \frac{[I]}{\alpha \cdot K_i} \right)$$

$$= \frac{K_M \cdot \left(1+\frac{[I]}{K_i}\right)}{v_{max}} \cdot \frac{1}{[S]} + \frac{1}{v_{max}} \cdot \left(1 + \frac{[I]}{\alpha \cdot K_i}\right) = m \cdot \frac{1}{[S]} + t \qquad (7)$$

For simplicity, the terms for the slope *m* and the ordinate shift *t* are combined in constants in the final expression. For all the species with inhibitory effects on PPO4 activity, the three straight lines obtained for the tested inhibitor concentrations intersected in one common point, respectively (see Figures S19-23A and 3A in the main text). The here observed location of the intersection points on neither axis of the coordinate system, but in the third quadrant, indicates a mixed-type inhibition behaviour. **$[PW_{12}]^{3-}$** and **$[WO_4]^{2-}$** gave very similar results, in line with the observations in the activity plots. As can be reasonably expected from structural analogy, the tested Keggin clusters show the same inhibition behaviour. The positive control kojic acid revealed a competitive inhibition type with all Lineweaver-Burk lines intersecting in a common point on the ordinate.

The slope *m* of the Lineweaver-Burk graphs allows for evaluation of K_i[30] when plotted against the inhibitor concentration *[I]*:

$$m = \frac{K_M \cdot \left(1+\frac{[I]}{K_i}\right)}{v_{max}} = \frac{K_M}{v_{max} \cdot K_i} \cdot [I] + \frac{K_M}{v_{max}} \qquad (8)$$

The straight lines obtained by fitting the three data points (*[I]*,*m*) for each tested inhibitor to equation (8) (see insets in Figures S19-23A and 3A in the main text) facilitate validation of the K_i values from the activity plots as they intersect the abscissa at a value of $-K_i$.

The ordinate intercepts *t* of the Lineweaver-Burk graphs can be evaluated for the inhibition parameter K_{is}[30], which corresponds to $\alpha \cdot K_i$ in Cope's nomenclature:

$$t = \frac{1+\frac{[I]}{\alpha \cdot K_i}}{v_{max}} = \frac{1}{v_{max} \cdot \alpha \cdot K_i} \cdot [I] + \frac{1}{v_{max}} \qquad (9)$$

With K_i known from the plots according to equation (8), another estimate for the parameter α can be extracted from the Lineweaver-Burk data.

3.2.2 Kinetic evaluation using Dixon plots

Finally, the inhibition mode was further assessed by Dixon plots[31]. Therefore, equation (7) was rearranged to obtain a linear dependence of $1/v_{app}$ on *[I]* for each of the five substrate concentrations used:

$$\frac{1}{v_{app}} = \frac{1}{v_{max}} \cdot \left(\frac{K_M \cdot \left(1+\frac{[I]}{K_i}\right)}{[S]} + 1 + \frac{[I]}{\alpha \cdot K_i} \right)$$

$$= \frac{1}{v_{max} \cdot K_i} \cdot \left(\frac{K_M}{[S]} + \frac{1}{\alpha}\right) \cdot [I] + \frac{1}{v_{max}} \cdot \left(1 + \frac{K_M}{[S]}\right) = m \cdot [I] + t \quad (10)$$

In a similar fashion as performed with the Lineweaver-Burk plots, the intersection behaviour of the Dixon lines is often used for determination of inhibition types. An ideal competitive inhibitor leads to an intersection point in the second quadrant, as demonstrated for kojic acid (see Figure S18B). The K_i value was verified from the found intersection point at ($-K_i$, v_{max}). Perfect non-competitive inhibition results in Dixon plots intersecting on the abscissa, and for uncompetitive inhibition parallel lines are obtained, which strongly suggests a mixed-type inhibition for the tungstate species as evident from the plots depicted here (Figures S19-23B and 3B in the main text). In accordance with the proposed mixed-type inhibition mode, the five lines obtained for each inhibitor did not intersect in a single common point, but rather within a common region. Nevertheless, we calculated an average intersection from the means of the intersections and used its x-coordinate as an estimate for $-K_i$ (cf. Table 1 in the main text). Fitting Dixon plots for mixed-type inhibition curves can be performed with non-linear equations[31] requiring more than three data points, which is why we used the presented linear simplification only to obtain a third estimate for the K_i value.

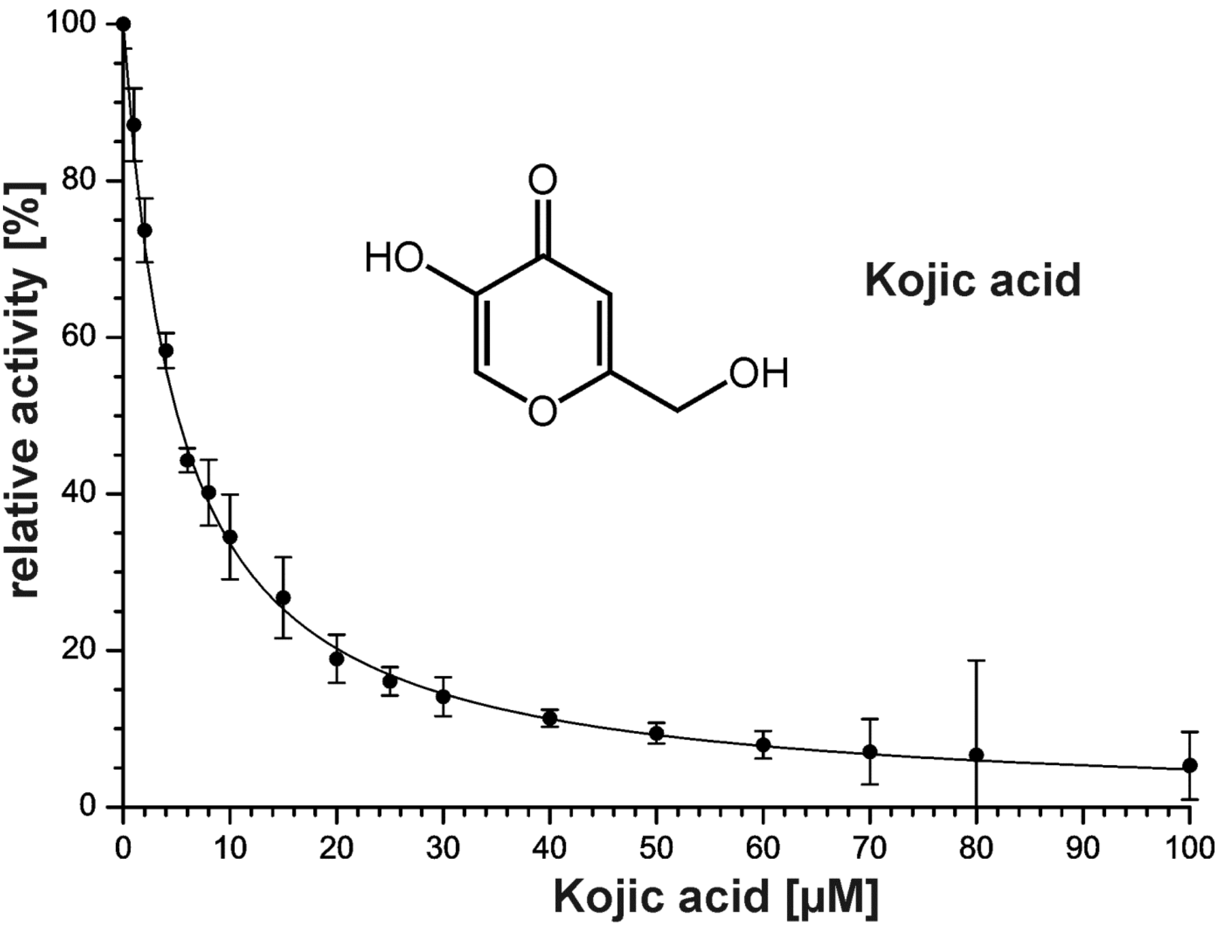

Figure S17. Activity plot for *ab*PPO4 inhibition by kojic acid. The dopachrome assay was performed with 1 mM *L*-DOPA (in 50 mM Na-citrate pH 6.8) and 0-14 mM POTs. The initial linear reaction rates were normalized with respect to the non-inhibited reaction velocity to be plotted as relative enzymatic activities and fitted according to equation (6). For fit parameters, see Table 1 in the main text.

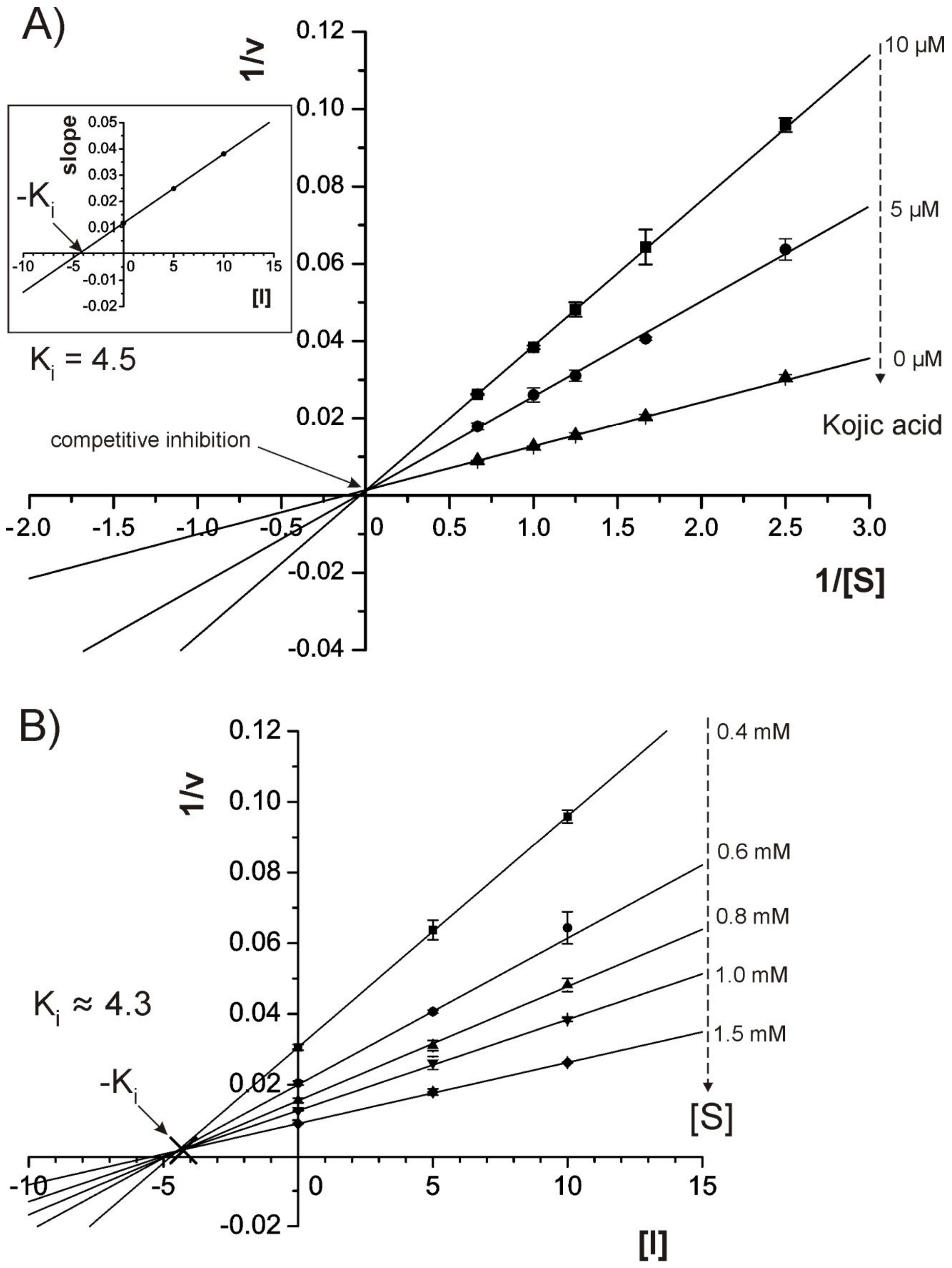

Figure S18. Kinetic evaluation of *ab*PPO4 inhibition by kojic acid using A) Lineweaver-Burk and B) Dixon plots. Fit parameters can be found in Table S3.

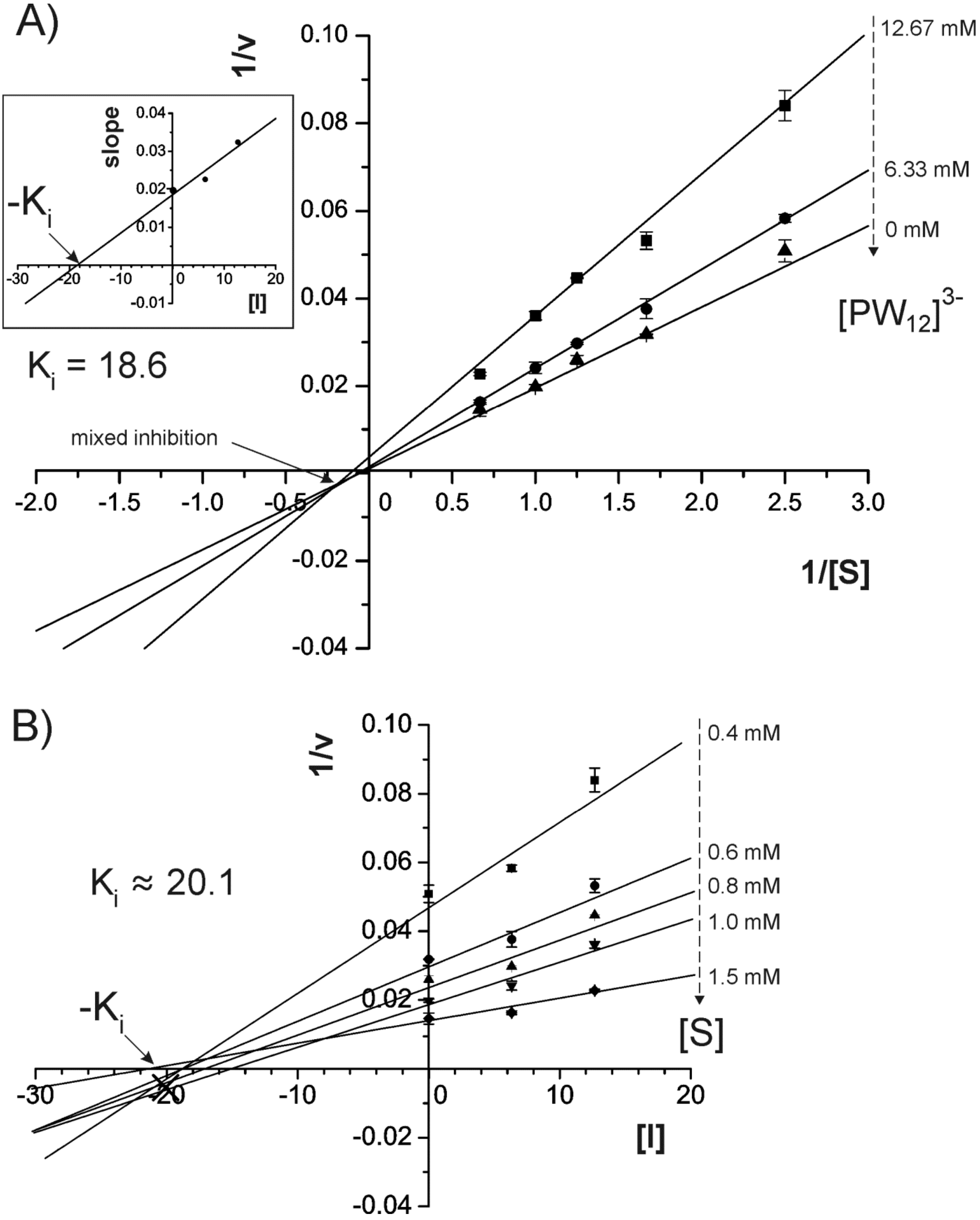

Figure S19. Kinetic evaluation of *ab*PPO4 inhibition by **$[PW_{12}]^{3-}$** using A) Lineweaver-Burk and B) Dixon plots. Fit parameters can be found in Table S3.

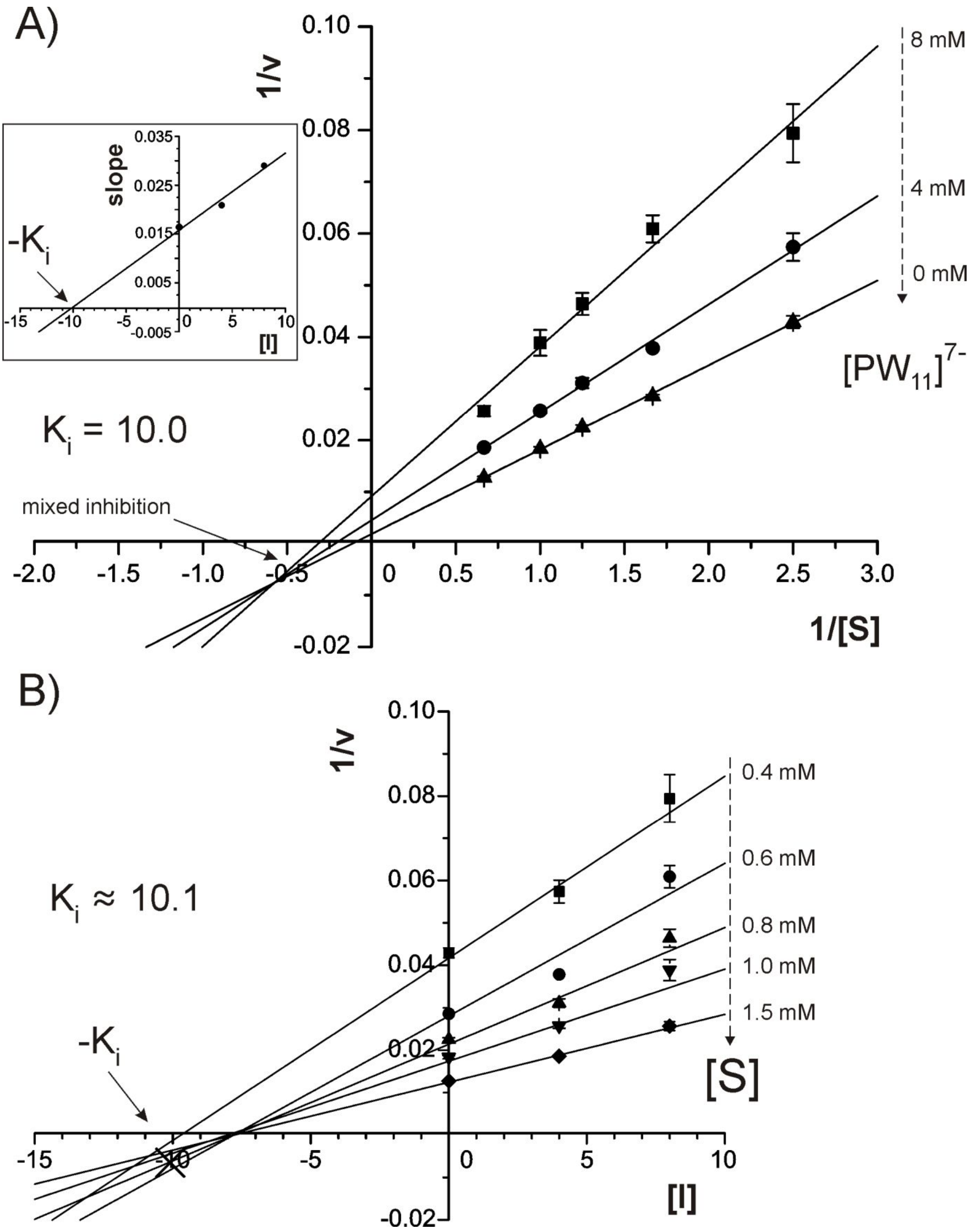

Figure S20. Kinetic evaluation of *ab*PPO4 inhibition by **$[PW_{11}]^{7-}$** using A) Lineweaver-Burk and B) Dixon plots. Fit parameters can be found in Table S3.

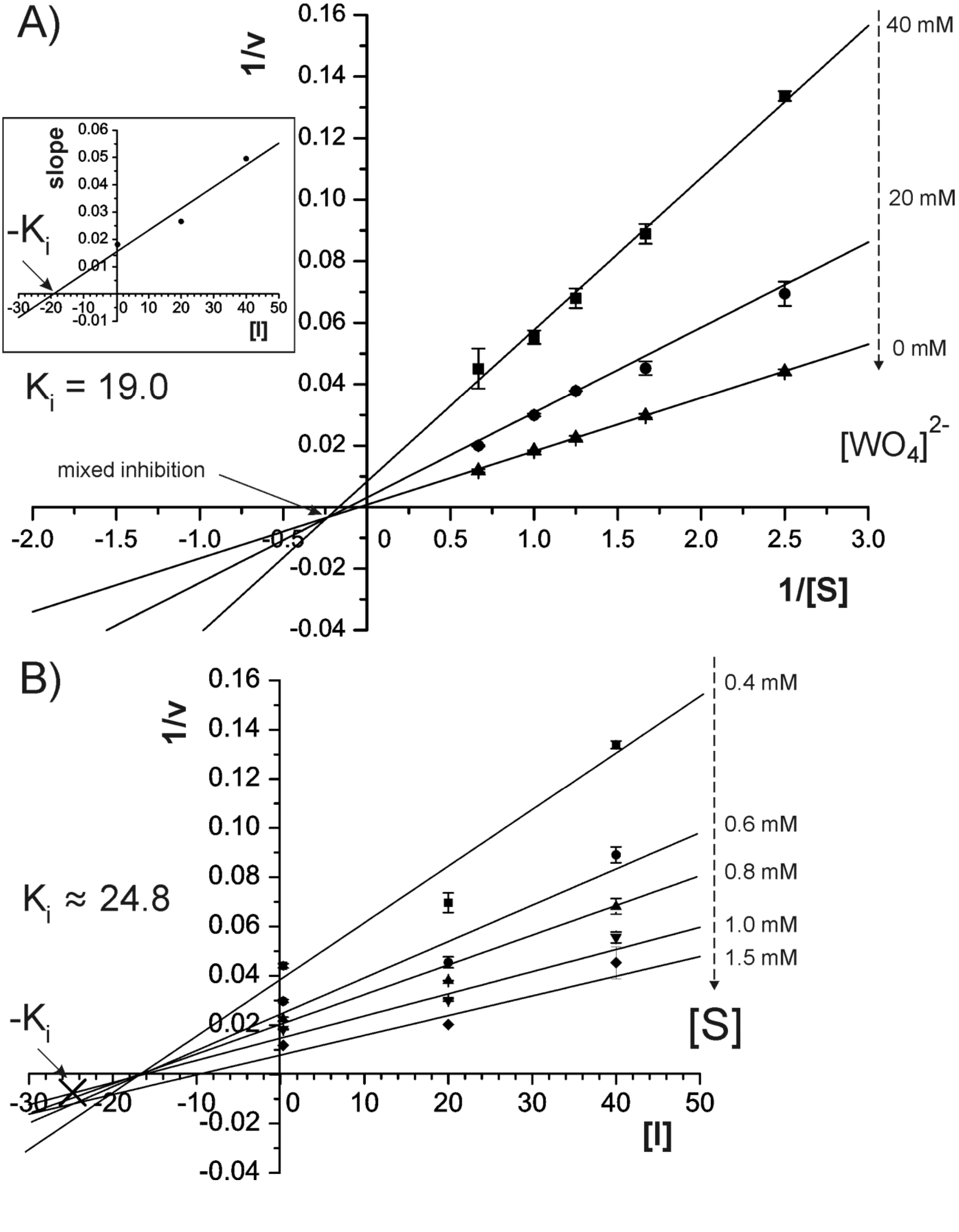

Figure S21. Kinetic evaluation of *ab*PPO4 inhibition by **$[WO_4]^{2-}$** using A) Lineweaver-Burk and B) Dixon plots. Fit parameters can be found in Table S3.

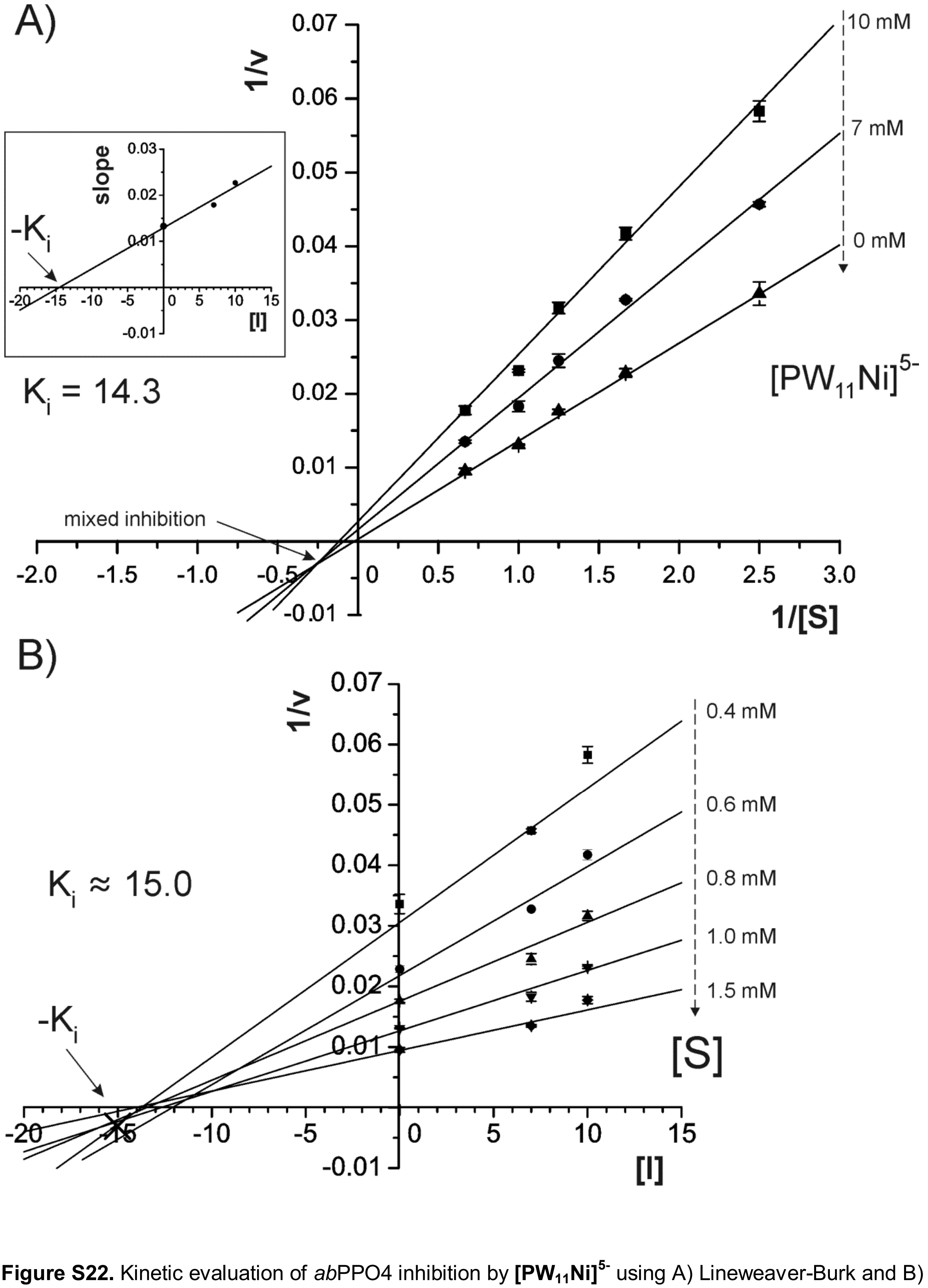

Figure S22. Kinetic evaluation of *ab*PPO4 inhibition by **$[PW_{11}Ni]^{5-}$** using A) Lineweaver-Burk and B) Dixon plots. Fit parameters can be found in Table S3.

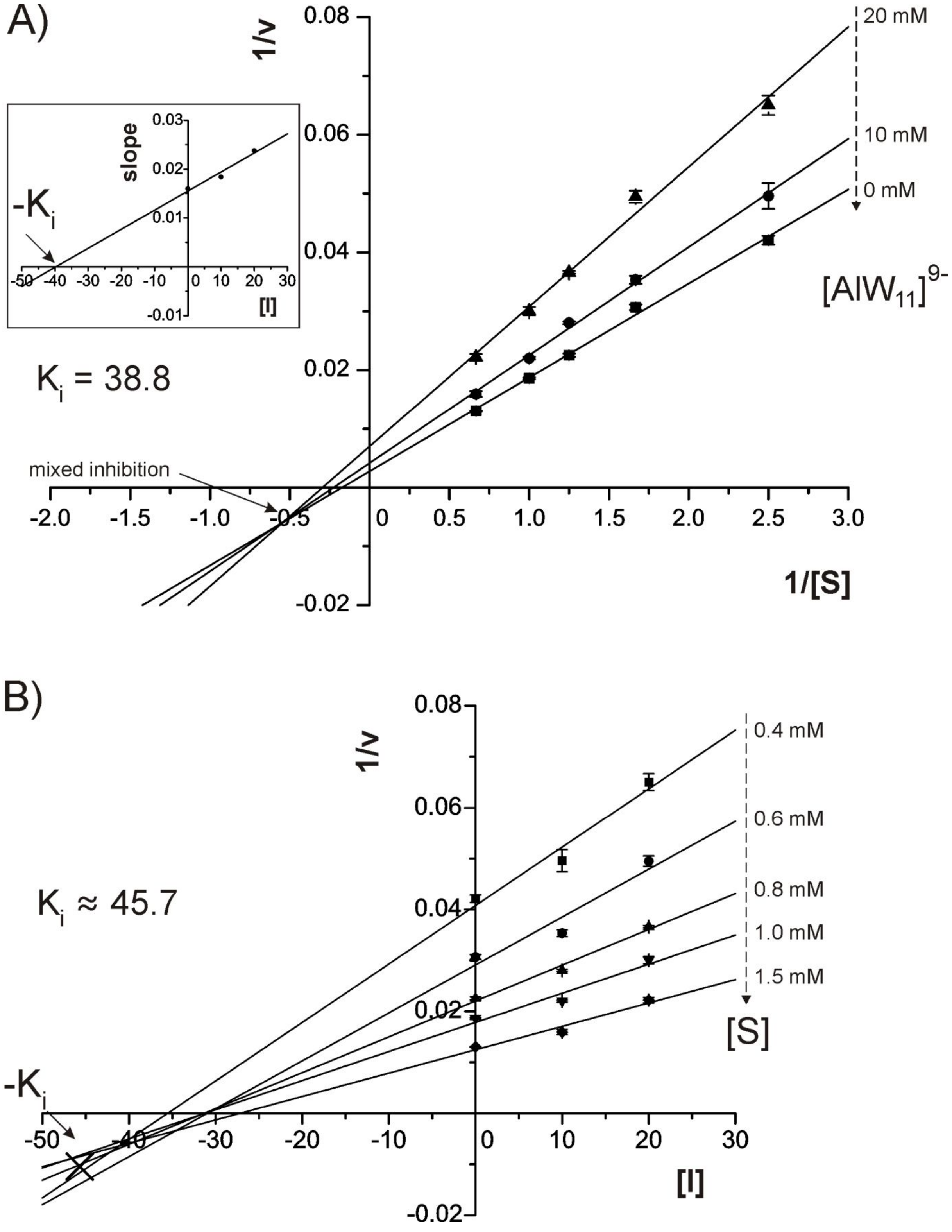

Figure S23. Kinetic evaluation of *ab*PPO4 inhibition by **$[AlW_{11}]^{9-}$** using A) Lineweaver-Burk and B) Dixon plots. Fit parameters can be found in Table S3.

Table S3. Parameters of straight line fits for kinetic evaluation according to Lineweaver-Burk and Dixon. *[I]*: inhibitor concentration, *[S]*: substrate concentration, *m*: straight line slope, *t*: straight line ordinate shift, R^2: line fit determination coefficient.

Inhibitor	Figure	*[I]*	*[S] [mM]*	*m*	*t*	R^2
**Kojic acid**	S18A	10 µM	0.4 - 1.5	0.038	0.0006	1.0
		5 µM		0.025	0.0007	1.0
		0 µM		0.012	0.0009	1.0
	Inset	0 - 10 µM		0.0026	0.012	1.0
	S18B	0 - 10 µM	0.4	0.0065	0.031	1.0
			0.6	0.0044	0.020	1.0
			0.8	0.0033	0.015	1.0
			1	0.0026	0.013	1.0
			1.5	0.0017	0.009	1.0
**$[PW_{12}]^{3-}$**	S19A	12.67 mM	0.4 - 1.5	0.032	0.0022	0.99
		6.33 mM		0.023	0.0011	1.0
		0 mM		0.020	0.0006	0.99
	Inset	0 - 12.67 mM		0.0010	0.019	0.91
	S19B	0 - 12.67 mM	0.4	0.0026	0.048	0.91
			0.6	0.0017	0.030	0.93
			0.8	0.0015	0.024	0.89
			1	0.0013	0.019	0.93
			1.5	0.0006	0.014	0.90
**$[PW_{11}]^{7-}$**	S20A	8 mM	0.4 - 1.5	0.029	0.0091	0.98
		4 mM		0.021	0.0045	1.0
		0 mM		0.016	0.0018	1.0
	Inset	0 - 8 mM		0.0016	0.016	0.97
	S20B	0 - 8 mM	0.4	0.0043	0.042	1.0
			0.6	0.0036	0.028	0.93
			0.8	0.0028	0.021	1.0
			1	0.0022	0.018	0.96
			1.5	0.0016	0.012	0.99
**$[WO_4]^{2-}$**	S21A	40 mM	0.4 - 1.5	0.049	0.008	0.99
		20 mM		0.026	0.003	0.99
		0 mM		0.018	0.0005	1.0
	Inset	0 - 40 mM		0.0008	0.015	0.94
	S21B	0 - 40 mM	0.4	0.0022	0.038	0.94
			0.6	0.0015	0.025	0.93
			0.8	0.0011	0.020	0.97
			1	0.0009	0.016	0.96
			1.5	0.0008	0.009	0.92
**$[SiW_{12}]^{4-}$**	3A (main text)	5 mM	0.4 - 1.5	0.039	0.012	0.98
		2.5 mM		0.028	0.007	0.99
		0 mM		0.017	0.0026	1.0
	Inset	0 - 5 mM		0.0045	0.017	1.0
	3B (main text)	0 - 5 mM	0.4	0.014	0.045	1.0
			0.6	0.0091	0.030	1.0
			0.8	0.0066	0.024	1.0
			1	0.0060	0.019	1.0
			1.5	0.0060	0.014	1.0
**$[PW_{11}Ni]^{5-}$**	S22A	10 mM	0.4 - 1.5	0.023	0.0024	0.99
		7 mM		0.018	0.0015	0.99
		0 mM		0.013	0.0005	1.0
	Inset	0 - 10 mM		0.0009	0.013	0.94
	S22B	0 - 10 mM	0.4	0.0023	0.033	0.94
			0.6	0.0018	0.022	0.96
			0.8	0.0013	0.017	0.95
			1	0.0010	0.013	0.96
			1.5	0.0008	0.009	0.94
**$[AlW_{11}]^{9-}$**	S23A	20 mM	0.4 - 1.5	0.024	0.007	0.99
		10 mM		0.018	0.0041	1.0
		0 mM		0.016	0.0027	1.0
	Inset	0 - 20 mM		0.0004	0.016	0.95
	S23B	0 - 20 mM	0.4	0.0011	0.041	0.96
			0.6	0.0009	0.029	0.92
			0.8	0.0007	0.022	0.98
			1	0.0006	0.018	0.95
			1.5	0.0005	0.013	0.96

Table S4. Parameters of straight line fits for kinetic evaluation of the Lineweaver-Burk ordinate intercepts to obtain $\alpha \cdot K_i$ estimates. *[I]*: inhibitor concentration, *[S]*: substrate concentration, *m*: straight line slope, *t*: straight line ordinate shift, R^2: line fit determination coefficient.

Inhibitor	$\alpha \cdot K_i$	*[I]*	*[S] [mM]*	*m*	*t*	R^2
$[PW_{12}]^{3-}$	3.8	0 - 12.67 mM	0.4 - 1.5	0.00013	0.0005	0.96
$[PW_{11}]^{7-}$	1.7	0 - 8 mM	0.4 - 1.5	0.00091	0.0015	0.98
$[WO_4]^{2-}$	0.5	0 - 40 mM	0.4 - 1.5	0.00019	0.0001	0.96
$[SiW_{12}]^{4-}$	1.2	0 - 5 mM	0.4 - 1.5	0.00193	0.0024	1.0
$[PW_{11}Ni]^{5-}$	2.8	0 - 10 mM	0.4 - 1.5	0.00018	0.0005	0.97
$[AlW_{11}]^{9-}$	11.9	0 - 20 mM	0.4 - 1.5	0.00021	0.0025	0.96

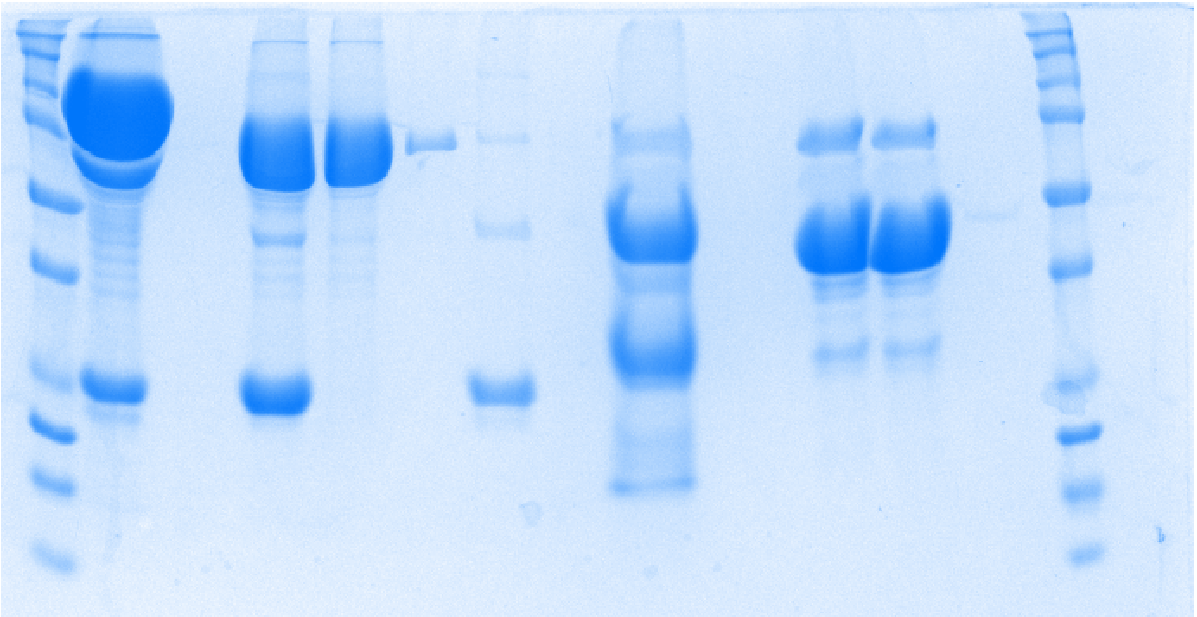

Figure S24. Original gel photography of the SDS-PAGE analysis shown in Figure S1. The image was taken with a Biorad Molecular Imager Gel Doc XR documentation system using the instrument software Image Lab (version 5.2.1) with standard settings for Coomassie-stained SDS-PAGE.

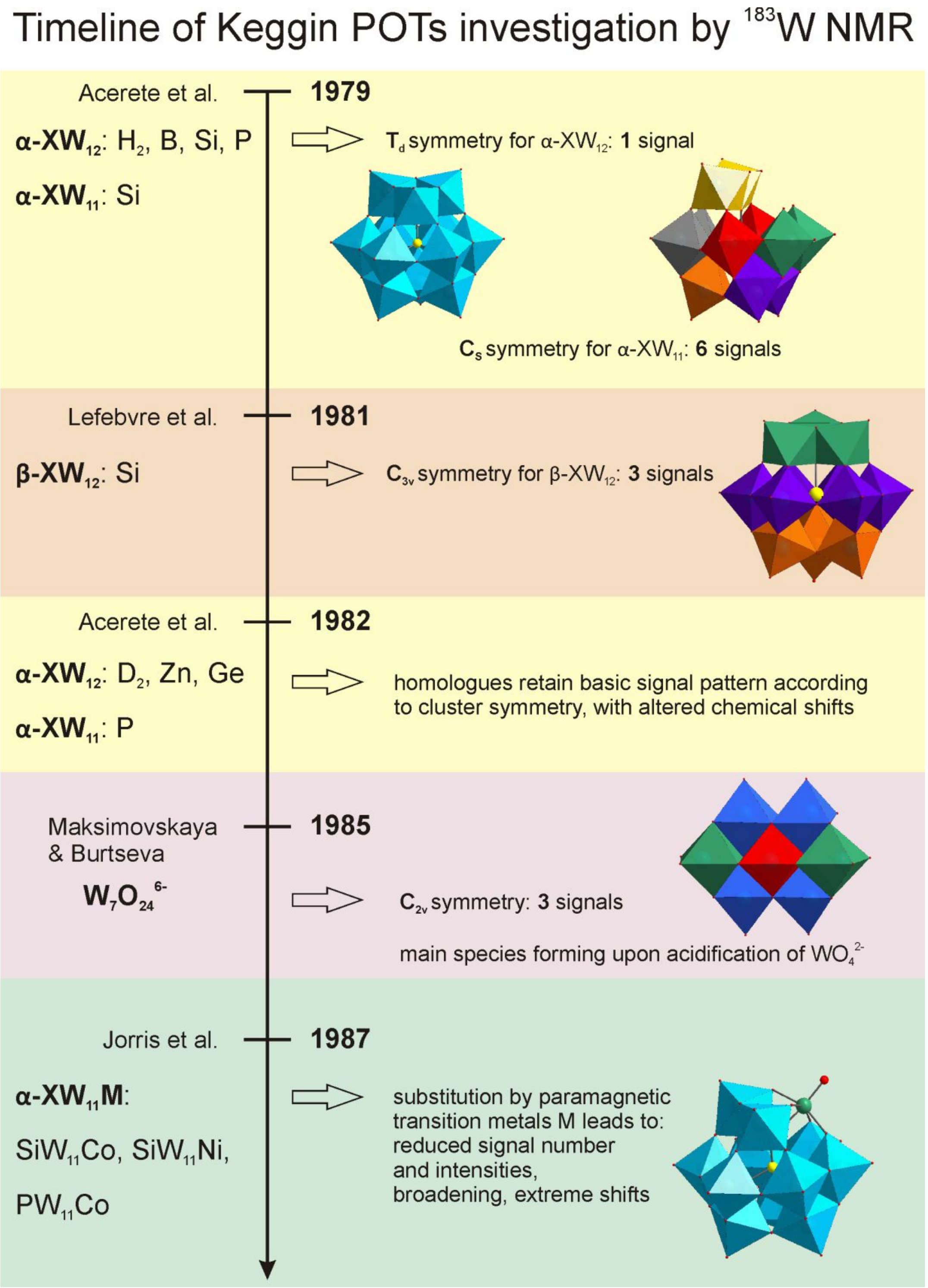

Figure S25. Timeline of selected milestones in the structural characterization of Keggin POTs by ^{183}W NMR. Since the 1980's, ^{183}W NMR analysis facilitates distinction of α- and β-isomers of the intact Keggin anion XW_{12}, the lacunary form α-XW_{11} and even monosubstituted clusters α-$XW_{11}M$ after insertion of a transition metal into the lacunary site. X depicts the tetrahedral heteroatom center of Keggin anions. Symmetry-equivalent W centers are depicted in the same colours in corresponding structure schemes. The Baker group started with the first NMR analysis of α-Keggin POTs in 1979 (Acerete *et al.* 1979 [10]). They extended their findings to homologous structures (Acerete *et al.* 1982 [7]) and transition metal substituted anions (Jorris *et al.* 1987 [21]). Lefebvre *et al.* (1981 [27]) contributed signal assignments to the β-Keggin isomer and Maksimovskaya & Burtseva (1985 [22]) identified the characteristic heptatungstate signal pattern which is abundant in acidified orthotungstate solutions.

4. Abbreviations

app as in v_{app}, $v_{max,app}$, $K_{M,app}$	apparent values upon enzymatic inhibition
α	inhibition parameter
[I]	inhibitor concentration
K_i	inhibition constant
K_M	Michaelis constant
m	slope of straight line
[S]	substrate concentration
v	actual reaction velocity
v_{max}	maximum reaction velocity
t	ordinate shift of straight line

5. References

[1] Pretzler, M., Bijelic, A. & Rompel, A. Heterologous expression and characterization of functional mushroom tyrosinase (*Ab*PPO4). *Sci. Rep.* **7,** 1810 (2017), DOI:10.1038/s41598-017-01813-1.

[2] Baker, L. C. W. & Figgis, J. S. A new fundamental type of inorganic complex: hybrid between heteropoly and conventional coordination complexes. Possibilities for geometrical isomerisms in 11-, 12-, 17-, and 18-heteropoly derivatives. *J. Am. Chem. Soc.* **92,** 3794-3797 (1970), DOI:10.1021/ja00715a047.

[3] Phillips, M. A. The preparation of phosphotungstic acid and of sodium and barium phosphotungstates. *J. Chem. Technol. Biotechnol.* **69**, 282-284 (1950), DOI:10.1002/jctb.5000690906.

[4] Keggin, J. F. Structure of the crystals of 12-phosphotungstic acid. *Nature* **131,** 351 (1933), DOI:10.1038/132351a0.

[5] Maksimovskaya, R. I. Hydrolysis of heteropoly acid $H_3PW_{12}O_{40}$ by ^{31}P NMR. *Russ. J. Inorg. Chem.* **43,** 1825-1837 (1998).

[6] Sveshnikov, N. N. & Pope, M. T. Assignment of multiline tungsten-183 NMR spectra of diamagnetic polyoxotungstates from intensity patterns**.** *Inorg. Chem.* **39,** 591-594 (2000), DOI:10.1021/ic9908928.

[7] Acerete, R., Hammer, C. F. & Baker, L. C. W. ^{183}W NMR of heteropoly- and isopolytungstates. Explanations of chemical shifts and band assignments. Theoretical considerations. *J. Am. Chem. Soc.* **104,** 5384-5390 (1982), DOI:10.1021/ja00384a023.

[8] Teaze, A., Herve, G., Finke, R. G. & Lyon, D. K. α-, β-, and g-Dodecatungstosilicic Acids: Isomers and Related Lacunary Compounds. In Inorganic Syntheses; Ginsberg, A. P., Ed.; John Wiley & Sons,Inc., **27**, 85–96 (1990).

[9] Akiko, K. & Yukiyoshi, S. The Crystal Structure of α-Barium 12-Tungstosilicate, α-$Ba_2SiW_{12}O_{40}\cdot 16H_2O$. *Bull. Chem. Soc. Jpn.*, **48**, 885-888 (1975), DOI:10.1246/bcsj.48.885.

[10] Acerete, R., Hammer, C. F. & Baker, L. C. W. Direct tungsten-183 nuclear magnetic resonance: a powerful new structural tool for heteropoly- and isopolytungstate chemistry. *J. Am. Chem. Soc.***101,** 267-269 (1979), DOI:10.1021/ja00495a070.

[11] Rocchiccioli-Deltcheff, C., Fournier, M., Franck, R. & Thouvenot, R. Vibrational investigations of polyoxometalates. 2. Evidence for anion-anion interactions in molybdenum(VI) and tungsten(VI) compounds related to the Keggin structure. *Inorg. Chem.* **22,** 207-216 (1983), DOI:10.1021/ic00144a006.

[12] Kraus, O. Untersuchungen über das Kristallgitter von Heteropolysäuren und deren Salzen. *Z. Kristallogr. Krist.* **94,** 256-279 (1936).

[13] Chen, Y.-G., Gong, J. & Qu, L.-Y. Tungsten-183 nuclear magnetic resonance spectroscopy in the study of polyoxometalates. *Coord. Chem. Rev*. **248**, 245–260 (2004). DOI:10.1016/j.cct.2003.11.003.
[14] Cowan, J. J., Hill, C. L., Reiner, R. S., Weinstock, I. A. Dodecatungstoaluminic acid and its monolacunary and mixed-addendum derivatives. *Inorg. Synth.* **33,** 18-25 (2002).
[15] Weinstock, I. A., Cowan, J. J., Barbuzzi, E. M. G., Zeng, H. & Hill, C. L. Equilibria between α and β isomers of Keggin heteropolytungstates. *J. Am. Chem. Soc.* **121,** 4608-4617 (1999), DOI:10.1021/ja982908j.
[16] Asami, M., Ichida, H. & Sasaki, Y. The structure of hexakis(tetramethylammonium) di-hydrogendodecatungstate enneahydrate, $[(CH_3)_4N]_6[H_2W_{12}O_{40}]\cdot 9H_2O$. *Acta Cryst. C* **40**, 35-37 (1984), DOI:10.1107/S0108270184002924.
[17] Santos, J. A. An X-ray study of the cæsium salts of certain 12-heteropoly acids. *Proc. Roy. Soc. A* **150,** 309-322 (1935), DOI:10.1098/rspa.1935.0103.
[18] Anyushin, A. V. *et al.* Synthesis and characterization of a new Keggin anion: $[BeW_{12}O_{40}]^{6-}$. *Chem. Commun.* **50,** 9083-9085 (2014), DOI:10.1039/C4CC02456G.
[19] Weakley, T. J. R. & Malik, S. A. Heteropolyanions containing two different heteroatoms – I. *J. Inorg. Nucl. Chem.* **29,** 2935-2944 (1967), DOI:10.1016/0022-1902(67)80126-X.
[20] Weakley, T. J. R. Crystal structure of cesium aquanickelo(II)undecatungstophosphate dihydrate. *J. Crystallogr. Spectrosc. Res.* **17,** 383–391 (1987).
[21] Jorris, T. L. *et al.* Effects of paramagnetic and diamagnetic transition-metal monosubstitutions on ^{183}W and ^{31}P NMR spectra for Keggin and Wells-Dawson heteropolytungstate derivatives. Correlations and corrections. ^{183}W NMR two-dimensional INADEQUATE studies of α-$[(D_2O)ZnO_4X^{n+}W_{11}O_{34}]^{(10-n)-}$ wherein $X^{n+} = Si^{4+}$ and P^{5+}. *J. Am. Chem. Soc.***109,** 7402-7408 (1987), DOI:10.1021/ja00258a025.
[22] Maksimovskaya, R. I. & Burtseva, K. G. ^{17}O and ^{183}W NMR studies of the paratungstate anions in aqueous solutions. *Polyhedron* **4**, 1559-1562 (1985), DOI: 10.1016/S0277-5387(00)87227-7.
[23] Fan, L., Cao, J. & Hu, C. What can electrospray mass spectrometry of paratungstates in an equilibrating mixture tell us? *RSC Adv.* **5,** 83377-83382 (2015), DOI:10.1039/C5RA18059G.
[24] Frenzel, R. A., Romanelli, G. P., Blanco, M. N. & Pizzio, L. R. Transition metal-modified polyoxometalates supported on carbon as catalyst in 2-(methylthio)-benzothiazole sulfoxidation. *J. Chem. Sc*i., **127**, 123-132 (2015). DOI:10.1007/s12039-014-0757-y.
[25] Fuchs, J. & Thiele, A & Palm, R. Struktur und Schwingungsspektrum des α-Undekawolframatophosphats $Na_2[N(CH_3)_4]_4HPW_{11}O_{39} \cdot 7H_2O$ / Structure and vibrational spectrum of the α-undecatungstophosphate $Na_2[N(CH_3)_4]_4HPW_{11}O_{39} \cdot 7H_2O$. *Z. Naturforsch. B* **36,** 544-550 (1981), DOI:10.1515/znb-1981-0504.
[26] Matsukoto, K. Y. & Sasaki; Y. The crystal structure of α-$K_8SiW_{11}O_{39}\cdot 13H_2O$. *Bull. Chem. Soc. Jpn.* **49,** 156-158 (1976), DOI:10.1246/bcsj.49.156.
[27] Lefebvre, J., Chauveau, F., Doppel, P., Brevard, C. ^{183}W NMR spectroscopy: $^{2}J_{w\text{-}w}$ coupling. Structural application to 1-12 heteropolytungstates. *J. Am. Chem. Soc.***103,** 4589–4591 (1981), DOI:10.1021/ja00405a055.
[28] Copeland, R. A. Evaluation of enzyme inhibitors in drug discovery. A guide for medicinal chemists and pharmacologists. *Methods Biochem. Anal.* **46**, 1–265 (2005), DOI: 10.1002/9781118540398.
[29] Lineweaver, H. & Burk, D. The determination of enzyme dissociation constants. *J. Am. Chem. Soc.* **56,** 658–666 (1934), DOI:10.1021/ja01318a036.
[30] Chai, W. M. *et al.* Condensed Tannins from Longan Bark as Inhibitor of Tyrosinase: Structure, Activity, and Mechanism. *J. Agric. Food Chem.* **66**, 908-917 (2018) DOI:10.1021/acs.jafc.7b05481.
[31] Butterworth, P. J. The use of Dixon plots to study enzyme inhibition. *Biochim. Biophys. Acta Enzymol.* **289,** 251-253 (1972), DOI:10.1016/0005-2744(72)90074-5.
